# Integrating novel culturing and culture‐independent methods to unveil soil fungal dark matter, functional guilds and environmental drivers across Robinson Ridge, East Antarctica

**DOI:** 10.1080/21501203.2025.2579297

**Published:** 2025-12-09

**Authors:** Nicole Benaud, Nathali Machado de Lima, Sin Yin Wong, Xabier Vázquez-Campos, Priyanka Rani Majumdar, Daniel Wilkins, Kate Montgomery, Merle Wiechers, Brett A. Summerell, Belinda C. Ferrari

**Affiliations:** a School of Biotechnology and Biomolecular Sciences, UNSW Sydney, Sydney, Australia; b Department of Climate Change, Energy, the Environment and Water, Environmental Stewardship Program, Australian Antarctic Division, Kingston, Australia; c Australian Institute of Botanical Science, Royal Botanic Gardens and Domain Trust, Sydney, Australia

**Keywords:** Soil, fungi, Antarctica, lichen, cold adaptation

## Abstract

Global fungal diversity is estimated to range in the millions, yet it remains severely underexplored, especially in extreme environments. Fewer than 5% of species have been officially identified and characterised. Estimates suggest that 70%–90% of soil fungi remain uncultivable, often referred to as ‘fungal dark taxa’. This study shows a comprehensive assessment of the fungal diversity in Robinson Ridge, East Antarctica using both culturing and non-culture dependent methods. The soil community was assessed by ITS gene sequencing of 93 subsoil and 93 surface soils collected along 3 × 300 m long transects. Strains were isolated from soil following enrichment cultivation under oligotrophic conditions supplemented with excess hydrogen gas. Landscape heterogeneity significantly influenced the composition, diversity, and functionality of the communities. A high relative abundance of lichen-associated fungi was recorded in areas with low levels of nutrients and moisture. One hundred and thirty fungal strains were isolated, mainly comprising the classes *Eurotiomycetes* and *Dothideomycetes*. A large proportion of fungal ASVs were not shared between techniques, with an overlap of only 1.9% of the total produced sequences. Our study underscores the value of integrating culture-dependent and independent approaches to gain a more comprehensive understanding of fungal distribution, diversity, and functional potential.

## GRAPHICAL ABSTRACT

**Figure:**
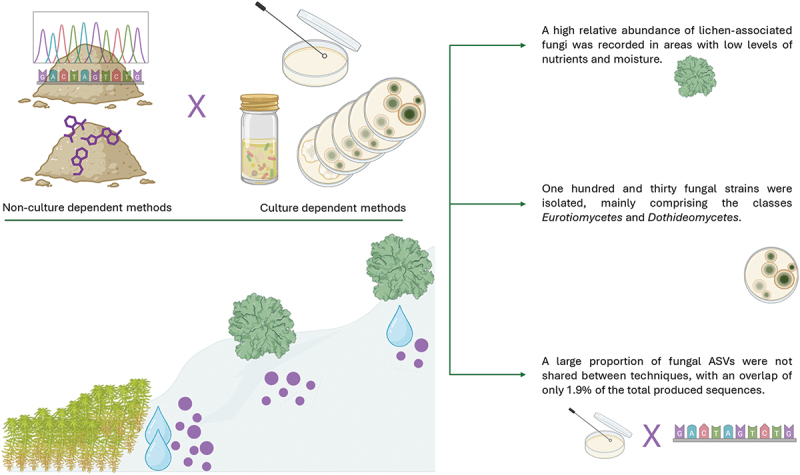

## 1. Introduction

Fungi are one of the most underexplored organisms on earth, with an estimated total number of species between 2.2 and 3.8 million (Hawksworth and Lücking 2017), and just 2.6%–4.5% of these are formally described (Hyde et al. 2020). Through environmental DNA sequencing, this large proportion of fungi that do not correspond to any known species and are yet to be cultured are known as “fungal dark taxa” (Ryberg and Nilsson 2018). Typically, only 0.1%–1% of microorganisms in an environmental sample are cultivable (Vartoukian et al. 2010; Solden et al. 2016), with estimates suggesting 70%–90% of soil fungi are yet-to-be cultured (Magnuson and Lasure 2002; Duarte et al. 2019). Extreme habitats contain an unexpectedly high degree of fungal species richness, including within rock substrates, deep oceans, volcanic and hot springs, acidic water bodies, and hot and cold deserts (Gonçalves et al. 2013, 2016; Wang 2017; de Menezes et al. 2020; Ogaki et al. 2020; Coleine et al. 2022; Segal-Kischinevzky et al. 2022).

Evidence suggests that fungi exhibit greater resilience than prokaryotes, enabling survival in inhospitable environments, compared with prokaryotes (Coleine et al. 2022). Their remarkable ecological adaptability is supported by physiological and morphological changes, including thickened and melanised cell walls, as well as the production of diverse secondary metabolites, such as UV protectant carotenoids and mycosporines (Gostinčar et al. 2012, 2022; Gonçalves et al. 2016). These adaptations enable them to withstand prolonged desiccation and intense solar radiation (Gonçalves et al. 2016; Coleine et al. 2022). In cold environments, such as the deserts of Antarctica, fungi tolerate high solar radiation, recurrent freeze-thaw cycles, osmotic stress, and a scarcity of water and nutrients (Ruisi et al. 2007; Obbels et al. 2016). Together with other microorganisms, fungi mediate the cycling of key biogeochemical elements such as nitrogen and carbon (Vero et al. 2019). Despite all this remarkable capacity, fungi remain one of the least explored components of the biota in the terrestrial Antarctica (Zucconi et al. 2024), with the culturable fraction lower than in temperate soils (Duarte et al. 2019). This gap represents a fundamental impediment to understanding the microbial ecology and biotechnological potential of fungi in cold deserts.

Most of our understanding of Antarctic fungal communities has been derived from studies on soil and rock samples collected from maritime Antarctica and the McMurdo Dry Valleys, while the majority of continental Antarctica has received less attention due to the difficulty of access (Doytchinov and Dimov 2022; Pushkareva et al. 2023). Advances in molecular phylogenetics and high-throughput DNA sequencing have enhanced our understanding of the Antarctic fungal life (Ji et al. 2016; Pudasaini et al. 2017; Zhang et al. 2020; Doytchinov and Dimov 2022; Rabelo et al. 2024). The studies have shown the dominance of *Ascomycota* followed by *Basidiomycota*, and investigations employing both culture-dependent and culture-independent methodologies have consistently demonstrated that microbial diversity recovered through cultivation is lower than that revealed by high-throughput sequencing techniques (Pudasaini et al. 2017; Selbmann et al. 2021). While cultivation-independent approaches recover only a small fraction of the total fungal diversity, certain taxa remain detectable exclusively through cultivation approaches (Selbmann et al. 2021). Consequently, a comprehensive understanding of microbial communities necessitates the integration of both culture-dependent and culture-independent strategies (Duarte et al. 2019).

Mimicking microbes’ natural environment and growth conditions have improved their cultivability (Kaeberlein et al. 2002). Novel micro-culturing techniques, such as “membrane diffusion-based cultivation” (Pudasaini et al. 2017; Benaud et al. 2022); “microfluidics-based cultivation methods” (Ge et al. 2016); and “cell sorting-based techniques” (Lee et al. 2019), have reduced the gap between culture-based and non-culture-based approaches. However, fungi have rarely been addressed (Rämä and Quandt 2021), with just a few studies using novel approaches (Delgado-Ramos et al. 2014; Uehling et al. 2019; Richter et al. 2022; Samlali et al. 2022; Li et al. 2023), and none of them were conducted in samples from Antarctica.

**Figure 1.**
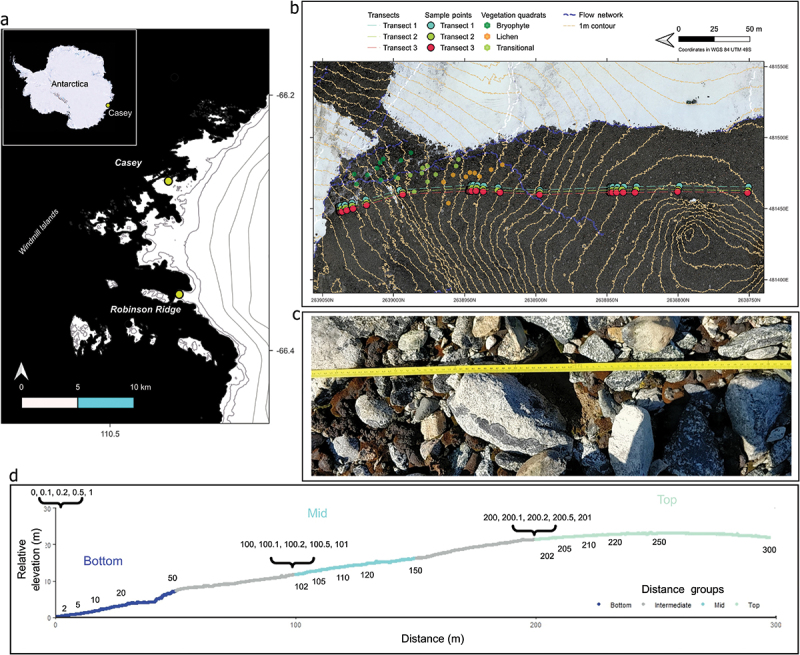
Location and geospatial sampling design of soils from Robinson Ridge, East Antarctica. (a) Location of Robinson Ridge and Casey station, Windmill Islands, and (inset) Casey station location in East Antarctica. (b) Drone orthomosaic of sampling transects showing vegetation, water flow, and slope. (c) Close up of soil and vegetation of the sample location used for culturing experiments (transect 1, distance 0 m). (d) Geospatial sampling design of collection points along transects (not to scale).

Robinson Ridge is a rocky, seasonally ice-free area located on a small peninsula near the coast of the Windmill Islands region of East Antarctica. The site is situated ~10 km South of the closest major Australian research facility, Casey station (Figure 1), and exhibits signs of climate-induced drying impacting vegetation communities (Robinson et al. 2018). In this region, previous studies have demonstrated that landscape connectedness significantly correlates with community structure and connectivity (Ferrari et al. 2016) and that fungi represent a substantial portion of the relative abundance within eukaryotic soil communities (Zhang et al. 2020). Soil fertility was shown to have a stronger influence on determining richness and evenness in the fungal communities of Robinson Ridge (Siciliano et al. 2014). Additionally, higher proportions of *Ascomycota* compared to *Basidiomycota* were observed in drier soils, and distinct communities were found in surface versus subsoil layers (Wong et al. 2025). Recently, our group reported *Penicillium psychrofluorescens* as a novel fungal species isolated from the Robinson Ridge site (Furnell et al. 2025). Many biosynthetic gene clusters were identified using genomic analysis, the majority of which are unrelated to known compound ‘BGCs’, underscoring the still unexplored biotechnological potential of Antarctic fungi.

This study extends the study of Wong et al. (2025) while incorporating a more detailed investigation of the fungi community in Robinson Ridge. Both culture-dependent and culture-independent approaches were applied to characterise the fungi community, while also investigating the influence of previously produced physicochemical data in the community composition and functionality. The study area and experimental design followed the framework described by Wong et al. (2025), analysing transects across a hillslope with wind-exposed arid soils near the top, characterised by a higher abundance of lichens; and snowmelt-sustained-moss beds at the bottom. Correlations between community composition and environmental and edaphic factors were also assessed to identify if the community structure varied alongside landform variation. We hypothesised that 1) culture-dependent and independent approach would target distinct fungal groups; 2) communities in the elevated areas of the transect would be more similar to each other than those at the bottom of the transect (based on earlier observations in the same area for bacteria, archaea, and micro-eukaryotes – Wong et al. 2025); 3) fungal functional traits would associate with variations in landform.

## 2. Materials and methods

### 2.1. Soil sample collection

Robinson Ridge soil samples were collected in March 2019 along three parallel transects, 300 m long and 2 m apart (Figure 1a–c); as described in Wong et al. (2025). From each transect, 31 samples were collected, and based on their distance and elevation, samples were classified into three distance groupings: “Bottom” soils (relative elevation < 8 m, distance between 0 and 50 m), “Mid” soils (relative elevation 10–16 m, distance between 100 and 150 m), and “Top” soils (relative elevation 20–23 m, distance between 200 and 300 m) (Figure 1d). Two separate depths were sampled for each location, obtaining a total of 186 soil samples, comprising 93 subsoils (3–10 cm depth) and 93 surface soils (0–3 cm depth). Samples were collected using sterile techniques, passed through a 4.75 mm sieve, and stored at −20 ºC, then −80 ºC upon arrival in our lab in UNSW Sydney, Australia, until further analysis.

### 2.2. Cultivation of fungi from soil

The culturing methodology selected for this study was chosen based on the fact that microbial growth under cold conditions is slow (Gostinčar et al. 2022). Also, prior empirical observations in our laboratory that were focused on isolating high-affinity hydrogen-oxidising bacteria used 60 ppmv of hydrogen (unpublished data), and yielded the unexpected growth of diverse slow growing fungi. Given its demonstrated effectiveness in recovering fungal organisms, we applied the same method in the present study, providing carbon limited media combined with hydrogen gas supplementation.

Fungi were previously isolated from bulk soil, collected from the first transect at 0 m distance (id: 174394, RR/T1/0, 66°22’3.864’‘S, 110°35’7.224’’E) (Figure 1c; Montgomery 2021). Briefly, cultures were obtained from an enrichment prepared from one gram of soil suspended in 30 mL of sterile Milli-Q water in a 110 mL glass serum vial, and crimp sealed with a Balch stopper. Vials were incubated at 10 °C, 100 r/min in darkness for a total of six months with an excess of hydrogen (~60 ppmv) maintained in the vial headspace over the duration of the incubation period. The enrichment was fluorescence-activated cell sorted (FACS) by size into 96-well plates, with each well containing 300 µL of liquid 0.1× RAVAN media, pH 5.5 (Watve et al. 2000), and incubated for further two months. Contents of each well were then spread-plated onto 0.1× RAVAN pH 5.5 agar plates and incubated at 10 °C for up to one year, with visible colonies sub-cultured to purity on 0.1× RAVAN pH 5.5 and potato dextrose broth (P6685, Sigma-Aldrich) agar plates. Light Microscopy was performed on selected strains using either an Olympus B×61 microscope with DP80 camera (Olympus, Australia) using bright field or differential interference contrast at 1,000× magnification, or an Olympus C×43 light microscope equipped with an EP50 digital camera and Lens at 400× magnification.

### 2.3. DNA extraction, PCR, Sanger sequencing, and taxonomic analysis of fungal isolates

Genomic DNA was extracted from pure fungal colonies using the DNeasy Plant Pro Kit (Qiagen, 69204) according to manufacturer instructions with the following exceptions: Lysis step was performed in 2 mL screw top vials containing 0.25 g each of 0.1 mm and 0.5 mm glass beads and homogenised using the FastPrep®-120 homogenisation instrument (MP Biomedicals, Irvine, CA, USA) for 1 min, on speed setting 4.5 m/s. The cells of certain fungal isolates exhibited considerable rigidity, necessitating homogenisation to be performed twice with a 5-minute interval following every 20 s of bead-beating. The initial centrifugation step after homogenisation was conducted at 16,000 × *g*, with the exception of one isolate, for which centrifugation occurred at 20,817 × *g* to obtain the pellet. The concentration and quality of DNA were evaluated using a NanoDrop ND-2000 Spectrophotometer and the ND-1000 v. 3.3.0 software (Coleman Technologies, Wilmington, DE, USA). The DNA was subsequently stored in sterile 1.5 mL microcentrifuge tubes at −20 °C until it was required for PCR.

Taxonomic identification of strains was performed based on Sanger sequencing of the internal transcribed spacer region (ITS) gene, amplified from gDNA using either primer set ITS1 (5’-TCCGTAGGTGAACCTGCGG-3’)/ITS4 (5’-TCCTCCGCTTATTGATATGC-3’) or ITS5 (5’-GGAAGTAAAAGTCGTAACAAGG-3’)/ITS4 (19) (Integrated DNA technologies, Singapore). Reaction mixtures contained 5 µL 5× Green *Gotaq*® Flexi Buffer (Promega, Madison, WI, USA), 2.5 mmol/L MgCl_2_ (Promega), 0.2 mmol/L each dNTP (Bioline), 0.1 mg/mL BSA (Sigma-Aldrich), 0.5 µmol/L each primer, 0.625 U of *GoTaq*® Hotstart DNA Polymerase (Promega), 2 µL of purified DNA template, and Ultrapure™ water to 25 µL (Invitrogen). PCRs were conducted in a Mastercycler® nexus G×2 PCR Thermal Cycler (Eppendorf), Amplification for the ITS1/ITS4 primer set involved an initial denaturation step of 3 min at 94 °C followed by 30 cycles consisting of denaturation at 94 °C for 1 min, the annealing at 55 °C for 30 s, an elongation step at 72 °C for 1 min, and the final extension step at 72 °C for 5 min. Amplification conditions for the ITS5/ITS4 set required for a few fungal isolates differed slightly: an initial denaturation at 95 °C for 3 min, followed by 35 cycles of denaturation at 95 °C for 30 s, annealing at 55 °C for 30 s, elongation at 72 °C for 30 s, and a final extension at 72 °C for 5 min. PCR amplification was confirmed via gel electrophoresis.

**Table 1.** Closest taxonomic identification for the 130 fungal isolates from Robinson Ridge soil.

Species hypothesis matching for isolated strain sequence (1.5% threshold)							UNITE taxonomy		
Isolate	PCR primer set	SH code	SH compound taxonomy	Phylum	Class	Unite code		Closest taxonomy match unite	Percentage
KMR21	ITS1/ITS4	SH0940214.10FU	*Ascomycota* sp.	*Ascomycota*	Ascomycota_cls_unspecified	OP581908		*Penicillium* sp.	100
KMR84	ITS1/ITS4	s47	*Ascomycota* ord unspecified	*Ascomycota*	Ascomycota_cls_unspecified	UDB02372981		*Oidiodendron* sp.	96.528
KMR145	ITS1/ITS4	SH0940214.10FU	*Ascomycota* sp.	*Ascomycota*	Ascomycota_cls_unspecified	OR098604		*Penicillium sumatraense*	99.824
KMR1	ITS1/ITS4	SH0929422.10FU	*Ramichloridium punctatum*	*Ascomycota*	*Dothideomycetes*	KU131679		*Ramichloridium punctatum*	100
KMR10	ITS5/ITS4	SH0968448.10FU	*Acrocalymma fici*	*Ascomycota*	*Dothideomycetes*	KP170619		*Acrocalymma fici*	98.842
KMR114	ITS1/ITS4	new_sh_in 4	*Dothideomycetes* ord unspecified	*Ascomycota*	*Dothideomycetes*	MK448255		*Ramichloridium punctatum*	95.076
KMR125	ITS1/ITS4	SH0942222.10FU	*Periconia verrucosa*	*Ascomycota*	*Dothideomycetes*	OR636301		*Periconia verrucosa*	100
KMR128	ITS1/ITS4	SH0962330.10FU	*Cladosporium herbarum*	*Ascomycota*	*Dothideomycetes*	OR760515		*Cladosporium delicatulum*	100
KMR129	ITS1/ITS4	SH0962330.10FU	*Cladosporium herbarum*	*Ascomycota*	*Dothideomycetes*	OR760515		*Cladosporium delicatulum*	100
KMR13	ITS1/ITS4	new_sh_in 4	*Dothideomycetes* ord unspecified	*Ascomycota*	*Dothideomycetes*	MK448255		*Ramichloridium punctatum*	95.076
KMR132	ITS1/ITS4	SH0962330.10FU	*Cladosporium herbarum*	*Ascomycota*	*Dothideomycetes*	OR752059		*Cladosporium cladosporioides*	100
KMR140	ITS1/ITS4	SH0962330.10FU	*Cladosporium herbarum*	*Ascomycota*	*Dothideomycetes*	OR752059		*Cladosporium cladosporioides*	100
KMR142	ITS1/ITS4	SH0962330.10FU	*Cladosporium herbarum*	*Ascomycota*	*Dothideomycetes*	OR752059		*Cladosporium cladosporioides*	100
KMR152	ITS1/ITS4	SH0962330.10FU	*Cladosporium herbarum*	*Ascomycota*	*Dothideomycetes*	OR760533		*Cladosporium halotolerans*	100
KMR156	ITS1/ITS4	SH0962330.10FU	*Cladosporium herbarum*	*Ascomycota*	*Dothideomycetes*	OR752059		*Cladosporium cladosporioides*	100
KMR163	ITS1/ITS4	SH0842905.10FU	*Acrodontium crateriforme*	*Ascomycota*	*Dothideomycetes*	KX287268		*Acrodontium crateriforme*	100
KMR165	ITS1/ITS4	SH0962330.10FU	*Cladosporium herbarum*	*Ascomycota*	*Dothideomycetes*	OR760515		*Cladosporium delicatulum*	100
KMR181	ITS1/ITS4	SH0972084.10FU	*Zymoseptoria verkleyi*	*Ascomycota*	*Dothideomycetes*	DQ068346		*Zymoseptoria* sp.	100
KMR193	ITS5/ITS4	SH1004868.10FU	*Rachicladosporium antarcticum*	*Ascomycota*	*Dothideomycetes*	MT236596		*Fungi* sp.	98.102
KMR23	ITS1/ITS4	SH0962330.10FU	*Cladosporium herbarum*	*Ascomycota*	*Dothideomycetes*	OR752059		*Cladosporium cladosporioides*	100
KMR3	ITS1/ITS4	SH0962330.10FU	*Cladosporium herbarum*	*Ascomycota*	*Dothideomycetes*	OR760532		*Cladosporium sphaerospermum*	100
KMR4	ITS1/ITS4	SH0962330.10FU	*Cladosporium herbarum*	*Ascomycota*	*Dothideomycetes*	OR760533		*Cladosporium halotolerans*	100
KMR5	ITS1/ITS4	SH0962330.10FU	*Cladosporium herbarum*	*Ascomycota*	*Dothideomycetes*	OR803391		*Cladosporium* sp.	100
KMR51	ITS5/ITS4	SH0962330.10FU	*Cladosporium herbarum*	*Ascomycota*	*Dothideomycetes*	OR752059		*Cladosporium cladosporioides*	100
KMR6	ITS1/ITS4	SH0962330.10FU	*Cladosporium herbarum*	*Ascomycota*	*Dothideomycetes*	OR803391		*Cladosporium* sp.	100
KMR60	ITS1/ITS4	SH0962330.10FU	*Cladosporium herbarum*	*Ascomycota*	*Dothideomycetes*	OR760515		*Cladosporium delicatulum*	100
KMR65	ITS1/ITS4	new_singleton_ins45	*Venturiales*	*Ascomycota*	*Dothideomycetes*	MH861516		*Fusicladium ramoconidii*	98.235
KMR77	ITS1/ITS4	SH0962330.10FU	*Cladosporium herbarum*	*Ascomycota*	*Dothideomycetes*	OR760532		*Cladosporium sphaerospermum*	100
KMR78	ITS1/ITS4	SH0962330.10FU	*Cladosporium herbarum*	*Ascomycota*	*Dothideomycetes*	OR760515		*Cladosporium delicatulum*	100
KMR81	ITS1/ITS4	SH0962330.10FU	*Cladosporium herbarum*	*Ascomycota*	*Dothideomycetes*	MZ568161		*Cladosporium aciculare*	100
KMR91	ITS1/ITS4	SH0962330.10FU	*Cladosporium herbarum*	*Ascomycota*	*Dothideomycetes*	OR752059		*Cladosporium cladosporioides*	100
KMR92	ITS5/ITS4	SH0842954.10FU	*Teratosphaeriaceae* sp.	*Ascomycota*	*Dothideomycetes*	MT236696		*Fungi* sp.	98.413
KMR93	ITS1/ITS4	SH0962330.10FU	*Cladosporium herbarum*	*Ascomycota*	*Dothideomycetes*	PP097798		*Cladosporium sphaerospermum*	100
KMR98	ITS1/ITS4	SH0962330.10FU	*Cladosporium herbarum*	*Ascomycota*	*Dothideomycetes*	OR752059		*Cladosporium cladosporioides*	100
KMR100	ITS1/ITS4	SH0939722.10FU	*Penicillium marthae-christenseniae*	*Ascomycota*	*Eurotiomycetes*	MF788210		*Penicillium* sp.	100
KMR102	ITS1/ITS4	SH0939673.10FU	*Penicillium spinulosum*	*Ascomycota*	*Eurotiomycetes*	OR499153		*Penicillium* sp.	99.739
KMR103	ITS1/ITS4	SH0939722.10FU	*Penicillium marthae-christenseniae*	*Ascomycota*	*Eurotiomycetes*	ON989623		*Penicillium catalonicum*	100
KMR104	ITS1/ITS4	SH0939673.10FU	*Penicillium spinulosum*	*Ascomycota*	*Eurotiomycetes*	OR499153		*Penicillium* sp.	100
KMR107	ITS1/ITS4	SH0885673.10FU	*Herpotrichiellaceae* sp.	*Ascomycota*	*Eurotiomycetes*	MH856518		*Rhinocladiella atrovirens*	99.77
KMR11	ITS1/ITS4	SH0888267.10FU	*Aspergillus monodii*	*Ascomycota*	*Eurotiomycetes*	OQ626373		*Aspergillus amoenus*	100
KMR115	ITS1/ITS4	SH0901348.10FU	*Talaromyces ramulosus*	*Ascomycota*	*Eurotiomycetes*	OW987178		*Talaromyces ramulosus*	98.981
KMR12	ITS1/ITS4	SH0888267.10FU	*Aspergillus monodii*	*Ascomycota*	*Eurotiomycetes*	OR760521		*Aspergillus versicolor*	100
KMR123	ITS1/ITS4	SH0939673.10FU	*Penicillium spinulosum*	*Ascomycota*	*Eurotiomycetes*	OP596060		*Penicillium glabrum*	100
KMR133	ITS1/ITS4	SH0888267.10FU	*Aspergillus monodii*	*Ascomycota*	*Eurotiomycetes*	AY373881		*Aspergillus versicolor*	100
KMR136	ITS1/ITS4	SH0901348.10FU	*Talaromyces ramulosus*	*Ascomycota*	*Eurotiomycetes*	FJ491787		*Talaromyces ramulosus*	99.616
KMR14	ITS1/ITS4	SH0888267.10FU	*Aspergillus monodii*	*Ascomycota*	*Eurotiomycetes*	OP179091		*Aspergillus puulaauensis*	99.815
KMR154	ITS1/ITS4	SH0939673.10FU	*Penicillium spinulosum*	*Ascomycota*	*Eurotiomycetes*	OR633247		*Penicillium glabrum*	100
KMR17	ITS1/ITS4	SH0939673.10FU	*Penicillium spinulosum*	*Ascomycota*	*Eurotiomycetes*	OR633247		*Penicillium glabrum*	100
KMR18	ITS5/ITS4	SH0767731.10FU	*Penicillium melinii*	*Ascomycota*	*Eurotiomycetes*	OW988079		*Penicillium citreonigrum*	100
KMR185	ITS1/ITS4	SH0939544.10FU	*Penicillium polonicum*	*Ascomycota*	*Eurotiomycetes*	OR983354		*Penicillium* sp.	100
KMR20	ITS1/ITS4	SH0801466.10FU	*Phaeomoniella* sp.	*Ascomycota*	*Eurotiomycetes*	GQ999270		*Phaeomoniella* sp.	98.621
KMR26	ITS1/ITS4	SH0767731.10FU	*Penicillium melinii*	*Ascomycota*	*Eurotiomycetes*	OQ297068		*Penicillium* sp.	99.821
KMR27	ITS1/ITS4	SH0767632.10FU	*Penicillium citrinum*	*Ascomycota*	*Eurotiomycetes*	OR764848		*Penicillium citrinum*	100
KMR29	ITS1/ITS4	SH0939673.10FU	*Penicillium spinulosum*	*Ascomycota*	*Eurotiomycetes*	OR633247		*Penicillium glabrum*	100
KMR30	ITS1/ITS4	SH0940222.10FU	*Penicillium roseopurpureum*	*Ascomycota*	*Eurotiomycetes*	OU989463		*Penicillium sanguifluum*	100
KMR32	ITS5/ITS4	SH0888267.10FU	*Aspergillus monodii*	*Ascomycota*	*Eurotiomycetes*	OW987951		*Aspergillus creber*	100
KMR33	ITS5/ITS4	SH0888267.10FU	*Aspergillus monodii*	*Ascomycota*	*Eurotiomycetes*	OW987951		*Aspergillus creber*	99.642
KMR44	ITS1/ITS4	SH0939544.10FU	*Penicillium polonicum*	*Ascomycota*	*Eurotiomycetes*	OR844310		*Penicillium rubens*	100
KMR50	ITS1/ITS4	SH0939673.10FU	*Penicillium spinulosum*	*Ascomycota*	*Eurotiomycetes*	MW221127		*Penicillium glabrum*	100
KMR52	ITS5/ITS4	SH0939544.10FU	*Penicillium polonicum*	*Ascomycota*	*Eurotiomycetes*	MK450710		*Penicillium raistrickii*	99.825
KMR53	ITS5/ITS4	SH0939673.10FU	*Penicillium spinulosum*	*Ascomycota*	*Eurotiomycetes*	ON229446		*Penicillium glabrum*	99.823
KMR54	ITS1/ITS4	SH0939673.10FU	*Penicillium spinulosum*	*Ascomycota*	*Eurotiomycetes*	OQ297072		*Penicillium* sp.	100
KMR63	ITS1/ITS4	SH0940222.10FU	*Penicillium roseopurpureum*	*Ascomycota*	*Eurotiomycetes*	OU989463		*Penicillium sanguifluum*	99.82
KMR64	ITS1/ITS4	SH0888267.10FU	*Aspergillus monodii*	*Ascomycota*	*Eurotiomycetes*	OR899610		*Aspergillus jensenii*	100
KMR66	ITS1/ITS4	SH0939673.10FU	*Penicillium spinulosum*	*Ascomycota*	*Eurotiomycetes*	OR673681		*Penicillium spinulosum*	100
KMR69	ITS1/ITS4	SH0939673.10FU	*Penicillium spinulosum*	*Ascomycota*	*Eurotiomycetes*	OP179016		*Penicillium glabrum*	100
KMR71	ITS1/ITS4	SH0940202.10FU	*Penicillium christenseniae*	*Ascomycota*	*Eurotiomycetes*	MH864114		*Penicillium* sp.	100
KMR73	ITS1/ITS4	SH0940683.10FU	*Penicillium* sp.	*Ascomycota*	*Eurotiomycetes*	OR884155		*Penicillium olsonii*	100
KMR74	ITS1/ITS4	SH0888267.10FU	*Aspergillus monodii*	*Ascomycota*	*Eurotiomycetes*	OP179091		*Aspergillus puulaauensis*	100
KMR76	ITS1/ITS4	SH0888267.10FU	*Aspergillus monodii*	*Ascomycota*	*Eurotiomycetes*	OR899610		*Aspergillus jensenii*	100
KMR8	ITS1/ITS4	SH0939673.10FU	*Penicillium spinulosum*	*Ascomycota*	*Eurotiomycetes*	OR673681		*Penicillium spinulosum*	100
KMR85	ITS1/ITS4	SH0939673.10FU	*Penicillium spinulosum*	*Ascomycota*	*Eurotiomycetes*	OR633247		*Penicillium glabrum*	100
KMR89	ITS1/ITS4	SH0888267.10FU	*Aspergillus monodii*	*Ascomycota*	*Eurotiomycetes*	OR808055		*Aspergillus* sp.	99.815
KMR97	ITS1/ITS4	SH0939673.10FU	*Penicillium spinulosum*	*Ascomycota*	*Eurotiomycetes*	ON229463		*Penicillium* sp.	100
KMR99	ITS1/ITS4	SH0939722.10FU	*Penicillium marthae-christenseniae*	*Ascomycota*	*Eurotiomycetes*	MF788210		*Penicillium* sp.	100
KMR141	ITS1/ITS4	new_singleton_in s35	*Leotiomycetes* ord unspecified	*Ascomycota*	*Leotiomycetes*	ON811506		*Ascocorticium sorbicola*	97.782
KMR15	ITS1/ITS4	SH0963782.10FU	*Geomyces auratus*	*Ascomycota*	*Leotiomycetes*	OR761536		*Pseudogymnoascus pannorum*	100
KMR16	ITS5/ITS4	SH0980625.10FU	*Pseudogymnoascus roseus*	*Ascomycota*	*Leotiomycetes*	PP125168		*Pseudogymnoascus pannorum*	100
KMR174	ITS1/ITS4	SH0950601.10FU	*Rutstroemiaceae* sp.	*Ascomycota*	*Leotiomycetes*	UDB06290687		*Rutstroemiaceae* sp.	99.363
KMR101	ITS5/ITS4	SH0925680.10FU	*Waltergamsia obpyriformis*	*Ascomycota*	*Sordariomycetes*	EF694655		*Sarocladium* sp.	99.808
KMR22	ITS1/ITS4	SH0758321.10FU	*Sarocladium implicatum*	*Ascomycota*	*Sordariomycetes*	MT102934		*Sarocladium implicatum*	99.825
KMR36	ITS1/ITS4	SH0740181.10FU	*Samsoniella hepiali*	*Ascomycota*	*Sordariomycetes*	MT974206		*Samsoniella alboaurantia*	100
KMR38	ITS1/ITS4	SH0982314.10FU	*Myrmecridium schulzeri*	*Ascomycota*	*Sordariomycetes*	MZ422994		*Myrmecridium schulzeri*	100
KMR41	ITS5/ITS4	SH0982314.10FU	*Myrmecridium schulzeri*	*Ascomycota*	*Sordariomycetes*	MZ422994		*Myrmecridium schulzeri*	100
KMR46	ITS1/ITS4	SH0884788.10FU	*Tilachlidium brachiatum*	*Ascomycota*	*Sordariomycetes*	KM190875		*Sordariomycetes* sp.	99.364
KMR55	ITS1/ITS4	SH0980683.10FU	*Sarocladium glaucum*	*Ascomycota*	*Sordariomycetes*	MN543910		*Sarocladium* sp.	99.451
KMR58	ITS1/ITS4	SH0898324.10FU	*Xylariales* sp.	*Ascomycota*	*Sordariomycetes*	OR760553		*Zygosporium masonii*	100
KMR70	ITS5/ITS4	SH0740128.10FU	*Lecanicillium saksenae*	*Ascomycota*	*Sordariomycetes*	OR752316		*Lecanicillium aphanocladii*	100
KMR80	ITS1/ITS4	SH0740145.10FU	*Cordycipitaceae* sp.	*Ascomycota*	*Sordariomycetes*	KU837817		*Fungi* sp.	100
KMR86	ITS1/ITS4	SH0982267.10FU	*Myrmecridium schulzeri*	*Ascomycota*	*Sordariomycetes*	MT138583		*Myrmecridium schulzeri*	99.541
KMR96	ITS5/ITS4	new_singleton_in s49	*Diaporthomycetidae* ord Incertae sedis	*Ascomycota*	*Sordariomycetes*	UDB06351972		*Rhamphoriaceae* sp.	93.46
KMR105	ITS5/ITS4	SH0896712.10FU	*Sebipora* sp.	*Basidiomycota*	*Agaricomycetes*	JN104571		*Sebipora* sp.	99.63
KMR118	ITS5/ITS4	SH1031006.10FU	*Fomitopsis* sp.	*Basidiomycota*	*Agaricomycetes*	ON994755		*Fomitopsis* sp.	99.358
KMR119	ITS1/ITS4	SH0881533.10FU	*Phlebia acanthocystis*	*Basidiomycota*	*Agaricomycetes*	KJ714010		*Phlebia acanthocystis*	99.833
KMR121	ITS1/ITS4	new_singleton_in s50	*Agaricomycetes* ord unspecified	*Basidiomycota*	*Agaricomycetes*	MK589284		*Nigroporus vinosus*	96.732
KMR122	ITS1/ITS4	SH0910764.10FU	*Aurantiporus fissilis*	*Basidiomycota*	*Agaricomycetes*	KU509495		*Bjerkandera atroalba*	99.543
KMR130	ITS1/ITS4	SH0831799.10FU	*Agaricomycetes* sp.	*Basidiomycota*	*Agaricomycetes*	OR262169		*Rigidoporus vinctus*	100
KMR143	ITS1/ITS4	new_singleton_in s36	*Agaricomycetes* ord unspecified	*Basidiomycota*	*Agaricomycetes*	MF399406		*Phanerochaete incarnata*	96.774
KMR157	ITS1/ITS4	SH0928352.10FU	*Bjerkandera adusta*	*Basidiomycota*	*Agaricomycetes*	ON682356		*Bjerkandera adusta*	99.664
KMR158	ITS1/ITS4	SH0938433.10FU	*Phlebia radiata*	*Basidiomycota*	*Agaricomycetes*	OP596501		*Phlebia acerina*	100
KMR161	ITS1/ITS4	SH0928335.10FU	*Bjerkandera fulgida*	*Basidiomycota*	*Agaricomycetes*	MW543001		*Bjerkandera adusta*	99.378
KMR164	ITS1/ITS4	SH1005272.10FU	*Sistotrema* sp.	*Basidiomycota*	*Agaricomycetes*	KY430527		*Sistotrema* sp.	99.842
KMR166	ITS5/ITS4	new_singleton_in s38	*Agaricomycetes* ord unspecified	*Basidiomycota*	*Agaricomycetes*	KU663313		*Fomitiporia neotropica*	84.06
KMR168	ITS1/ITS4	SH0896712.10FU	*Sebipora* sp.	*Basidiomycota*	*Agaricomycetes*	JN104571		*Sebipora* sp.	99.448
KMR170	ITS1/ITS4	SH0896712.10FU	*Sebipora* sp.	*Basidiomycota*	*Agaricomycetes*	JN104571		*Sebipora* sp.	99.263
KMR176	ITS1/ITS4	new_singleton_in s39	*Agaricomycetes* ord unspecified	*Basidiomycota*	*Agaricomycetes*	KP135056		*Meruliopsis* sp.	98.113
KMR2	ITS1/ITS4	SH0762233.10FU	*Polyporaceae* sp.	*Basidiomycota*	*Agaricomycetes*	MZ828067		*Trametes coccinea*	99.834
KMR24	ITS1/ITS4	SH0938433.10FU	*Phlebia radiata*	*Basidiomycota*	*Agaricomycetes*	OP596501		*Phlebia acerina*	100
KMR25	ITS1/ITS4	new_singleton_in s42	*Agaricomycetes* ord unspecified	*Basidiomycota*	*Agaricomycetes*	MT428549		*Pseudolagarobasidium baiyunshanense*	98.316
KMR56	ITS1/ITS4	SH0762233.10FU	*Polyporaceae* sp.	*Basidiomycota*	*Agaricomycetes*	FJ873395		*Pycnoporus coccineus*	99.671
KMR57	ITS1/ITS4	SH1024885.10FU	*Phanerochaete cumulodentata*	*Basidiomycota*	*Agaricomycetes*	OM716871		*Macrocybe gigantea*	99.836
KMR62	ITS1/ITS4	new_singleton_in s44	*Hymenochaetales*	*Basidiomycota*	*Agaricomycetes*	MT537076		*Skvortzovia meridionalis*	92.773
KMR88	ITS1/ITS4	SH0824122.10FU	*Agaricomycetes* sp.	*Basidiomycota*	*Agaricomycetes*	MK953245		*Coronicium alboglaucum*	100
KMR90	ITS1/ITS4	new_singleton_in s48	*Agaricomycetes* ord unspecified	*Basidiomycota*	*Agaricomycetes*	UDB02236172		*Irpicaceae* sp.	96.625
KMR159	ITS1/ITS4	new_singleton_in s37	*Cystobasidiomycetes* ord unspecified	*Basidiomycota*	*Cystobasidiomycetes*	AB520305		*Sporobolomyces* sp.	92.079
KMR153	ITS1/ITS4	SH0884506.10FU	*Robbauera albescens*	*Basidiomycota*	*Exobasidiomycetes*	OW982850		*Robbauera albescens*	99.686
KMR173	ITS1/ITS4	SH0884506.10FU	*Robbauera albescens*	*Basidiomycota*	*Exobasidiomycetes*	OW982850		*Robbauera albescens*	99.843
KMR177	ITS1/ITS4	SH0888474.10FU	*Gjaerumia minor*	*Basidiomycota*	*Exobasidiomycetes*	OW988226		*Gjaerumia minor*	99.815
KMR47	ITS5/ITS4	SH0888474.10FU	*Gjaerumia minor*	*Basidiomycota*	*Exobasidiomycetes*	AB025702		*Gjaerumia minor*	97.266
KMR72	ITS1/ITS4	SH0970589.10FU	*Quambalaria cyanescens*	*Basidiomycota*	*Exobasidiomycetes*	OW983127		*Quambalaria cyanescens*	100
KMR148	ITS1/ITS4	SH0911875.10FU	*Sporobolomyces ruberrimus*	*Basidiomycota*	*Microbotryomycetes*	OR803065		*Sporobolomyces* sp.	100
KMR68	ITS1/ITS4	SH0884346.10FU	*Fellomyces penicillatus*	*Basidiomycota*	*Tremellomycetes*	KY103409		*Fellomyces penicillatus*	99.803
KMR34	ITS1/ITS4	new_singleton_in s43	Eukaryota kgd Incertae sedis ord unspecified	Eukaryota_ kgd_Incertae_sedis_ phy_unspecified	Eukaryota_kgd_ Incertae_sedis_cls_ unspecified	OP584642		*Penicillium* sp.	99.638
KMR83	ITS5/ITS4	new_singleton_in s46	Eukaryota kgd Incertae sedis ord unspecified	Eukaryota_ kgd_Incertae_sedis_ phy_unspecified	Eukaryota_kgd_ Incertae_sedis_cls_ unspecified	LC015668		*Acremonium* sp.	93.058
KMR19	ITS5/ITS4	new_singleton_in s41	*Fungi* ord unspecified	Fungi_phy_ unspecified	Fungi_cls_unspecified	UDB0749392		*Fungi* sp.	90.485
KMR67	ITS1/ITS4	SH0740285.10FU	*Fungi* sp.	Fungi_phy_ unspecified	Fungi_cls_unspecified	OR760530		*Parengyodontium album*	100
KMR182	ITS1/ITS4	new_singleton_in s40	Fungi ord unspecified	Fungi_phy_ unspecified	Fungi_cls_unspecified	KC866431		*Phlebia* sp.	93.76
KMR116	ITS1/ITS4	NA	NA	NA	NA	OR076085		*Sympodiomycopsis paphiopedili*	99.798
KMR7	ITS1/ITS4	NA	NA	NA	NA	OR866089		*Blastobotrys* sp.	100
KMR106	ITS1/ITS4	NA	NA	NA	NA	MH856518		*Rhinocladiella atrovirens*	99.756

Evaluated from ITS sequences using both UNITE (UDB) and Protax SH species hypothesis matching of PCR amplified ITS regions.

PCR products were submitted to The Ramaciotti Centre for Gene Function Analysis, at UNSW Sydney (NSW, Australia), for purification and preparation of single-end sequencing of both forward and reverse primer regions, on the Sanger ABI 3730 Capillary Sequencer (Applied Biosystems, Scoresby, Australia). Resulting sequences were visualised with FinchTV v1.4.0 trace viewer (Geospiza, Seattle, WA, USA), quality trimmed to and merged. Resultant ITS gene regions (430–689 bp) were imported into the PlutoF platform (https://plutof.ut.ee/) (20) and analysed by SH Matching (v2.0.0, unprocessed), and also analysed by massBLASTer (BLAST + 2.16.0 – Camacho et al. 2009) against the UNITE reference sequence database v10 (Kõljalg et al. 2020; Abarenkov et al. 2022, 2024). The highest scoring matches from the UNITE analysis were reported alongside SH-matching at 1.5% clustering dissimilarity threshold. In the case of potential chimeras, we carefully checked the generated sequencing chromatograms generated by Sanger method and used the output from massBLASTer to check UNITE matches in detail.

### 2.4. Soil DNA extraction and ITS metabarcoding

The same soil samples used in Wong et al. (2025) were investigated here, now with specific focus on the fungal community. Total DNA from subsoil and surface samples (*n* = 186) (0.4 g) was extracted in triplicate using the FASTDNA™ SPIN Kit for Soil (MP Biomedicals, Seven Hills, NSW, Australia), and quantified using Quant-iT Picogreen dsDNA Assay kit (Life Technologies, VIC, Australia). The pool of each triplicate was subjected to paired-end amplicon sequencing of the internal transcribed spacer region (ITS) using primer set ITS1/ITS4 and sequenced in a MiSeq platform (Illumina) using the MiSeq Reagent kit v3 (2 × 300 bp) at The Ramaciotti Centre for Genomics. Raw amplicon reads are available at the Australian Antarctic Data Centre (Wong et al. 2025).

Raw sequence reads were processed into amplicon sequence variants (ASV) with DADA2 v1.30.0 (Callahan et al. 2016) in R v4.3.1 (R Core Team 2021). Briefly, reads with ambiguous bases were discarded. Residual primers were removed with cutadapt v4.3 (Martin 2011). Reads were filtered with the following parameters: reads matching phiX were removed, three maximum expected errors, minimum read length of 50 nt, and a quality threshold for read truncation of 2. Due to the extremely low proportion of paired reads able to be merged with mergePairs(), regardless of the trimming and/or merging parameters tested, only forward reads were processed further. Chimeric reads were removed with the removeBimeraDenovo() function with the consensus method. Non-chimeric reads were then collapsed with CD-HIT-EST v4.8.1 at 100% identity (Li and Godzik 2006). Taxonomy was assigned to merged reads with the assignTaxonomy() command, which implements the RDP Naive Bayesian Classifier algorithm (Wang et al. 2007) using the dynamically clustered UNITE general FASTA release for eukaryotes 10.0 (Abarenkov et al. 2024).

### 2.5. Soil physical and chemical analyses

Soil environmental data produced in Wong et al. (2025) were considered in this study, encompassing a total of 65 physical and chemical parameters from all the subsoil samples (*n* = 93, soil depth = 3–10 cm). This data was accessed through the Australia Antarctic Data Centre (https://data.aad.gov.au/metadata/AAS_4406_Robinson_Ridge_2019_DNA) and included Northing, Easting, height, aspect, slope, Terrain Roughness Index (TRI), distance from coast, moisture (1 - dry matter fraction), pH, conductivity, water-extractable ions (Cl^−^, NO_2_^−^, Br^−^, NO_3_^−^, PO_4_^3-^, SO_4_^2-^, and NH_3_), major and trace elemental oxides (SiO_2_, TiO_2_, Al_2_O_3_, Fe_2_O_3_, MgO, CaO, Na_2_O, K_2_O, P_2_O_5_, SO_3_, F, Cl, V_2_O_5_, Cr_2_O_3_, Mn_3_O_4_, NiO, CuO, ZnO, SrO, ZrO_2_, BaO), total chloride, fluoride, phosphorus, nitrogen, carbon, hydrogen, sulphur, and nutrient ions (P, K, Ca, Mg, Zn, B, S, Cu, Fe, Mn, Na) relative to the effective cation exchange capacity (eCEC).

### 2.6. ITS fungal richness, diversity, functionality, and distribution in Robinson Ridge soils

ANOVA combined with Tukey’s HSD post hoc tests was used to evaluate the physicochemical differences among the three positional soil groups (“Bottom”, “Mid”, and “Top”) as described in Wong et al. (2025). Relative abundances were calculated by sample using the tidyverse package v2.0.0 (Wickham et al. 2019) in R v4.1.2. Mean and median abundances were calculated for each taxon level according to transect distance groupings for “Bottom”, “Mid”, and “Top”. Soil taxonomy results were visualised using ggplot2 v3.4.4 (Wickham 2016). For phylum and class levels, relative abundance per sample was depicted as jitter plots. For order, family, genus, and species levels, ASV taxonomy was grouped as either the “known” (the taxonomy for that ASV was classifiable to that taxonomic level) and “unknown” (unclassifiable), and visualised as stacked bar charts. A Phyloseq object was generated in R v4.3.1 using the phyloseq package v1.44.0 (McMurdie and Holmes 2013), then relative abundance plots were run using tax_glom function; and DESeq2 v1.40.2 was used to determine differentially abundant taxa between different soil positional groups (Love et al. 2014).

For alpha diversity, ASV counts were randomly rarefied to an even depth of 10,000 reads using the rrarefy function from the vegan package v2.6-4 (Oksanen 2015). Following rarefaction, two low-count samples were removed from further alpha diversity analyses (RR/T2/101/Mid/B and RR/T1/100.2/Mid/B), leaving 184 total samples in the dataset. Alpha diversity metrics were computed on the rarefied data using the vegan package in R, for both species richness (S), and diversity using the Shannon-Wiener index. To determine whether differences between groups were statistically significant, analyses of variance were performed both on transect distance groupings (Bottom, Mid, Top) and soil layer (Subsoil, Surface), followed by post-hoc Tukey’s Honest Significant Difference test on transect distances.

Bray-Curtis dissimilarity matrices, derived from Hellinger-transformed ASV tables, were used for principal coordinates analysis (PCoA) for all 186 samples, and distance-based redundancy analysis (dbRDA), conducted in R 4.1.2 with the vegan package v2.6-4 on the 93 subsoil samples. Environmental variables analysed from the 93 subsoil samples, primarily log- or log(x + 1)-transformed (as detailed in Table S1), were tested for their correlation with microbial community structure using variance inflation factors (VIF) followed by forward model selection through permutation tests via the ordiR2step function. Permuted ANOVA with 999 permutations was applied to determine the significance of variables. Final dbRDA analysis was performed using statistically significant (*p* < 0.05) environmental variables Relative elevation (Height m), Distance from coast (m), ammonia (NH_3_ mg/kg DMB), nitrate (NO_3_^−^ mg/kg DMB), aluminium oxide Al_2_O_3_ (%), northing (m), and water extractable chloride (Cl^−^ mg/kg DMB). PCoA and dbRDA plots were visualised using ggplot2 v3.4.2.

To assign functionality for the whole community, UNITE’s assignment for each ASV at the genus level was cross-referenced with the FungalTraits database (Põlme et al. 2020). Their primary lifestyle abundance was calculated for presentation with a special focus on the 18 most abundant ASVs. Further analysis involved the use of Kruskal-Wallis test to check for significant correlations between the different functionalities and location. Spearman correlations were calculated using the microbiome R package to assess relationships between community composition and individual environmental variables. To identify which of the variables from Wong et al. (2025) were significantly associated with community structure, we applied the ordiR2step function from the vegan package v2.6-4 (Oksanen 2015), which performs stepwise model selection based on permutation tests within constrained ordination frameworks such as RDA. This was followed by a redundancy analysis to visualise and interpret the interactions between selected variables and microbial functionality.

### 2.7. Sequence alignment and taxonomic overlap

To evaluate the overlap between amplicon sequence variants (ASVs) and cultured isolate sequences we used blastn function from blast-plus v2.16 (Altschul et al. 1990) to search sequences from cultures versus ASVs produced by metabarcoding. Results were filtered to retain matches with a minimum of 99% sequence identity and where the alignment length covered at least 85% of the ASVs, ensuring near-complete and highly similar alignments. Libraries VennDiagram v1.7.3 (Chen and Boutros 2011), ggvenn v0.1.10 (Yan 2023), and ggplot2 v3.5.1 were used for all graphical representations.

## 3. Results

### 3.1. Fungal soil community diversity

A total of 12,784,999 ITS non-chimeric quality-filtered reads were recovered after processing the raw datasets from total soil gDNA, corresponding to a total of 2,600 ASVs assigned to the kingdom *Fungi*. In alpha diversity analyses on the randomly rarefied fungal community, the lowest average richness (88.5) was observed at the “Mid”-range of the transect groups followed by the “Bottom” (92.9) then “Top” (104.9). These differences were significant (*p* < 0.05), when the “Top” group was compared with both the “Mid” and “Bottom” of transects while the difference between the “Bottom” and “Mid” groups was not significant (*p* > 0.9). Surface soils exhibited a higher average richness (102.2) than Subsoils (89.5). However, this difference was not significant (*F* = 0, *p* > 0.9).

**Figure 2.**
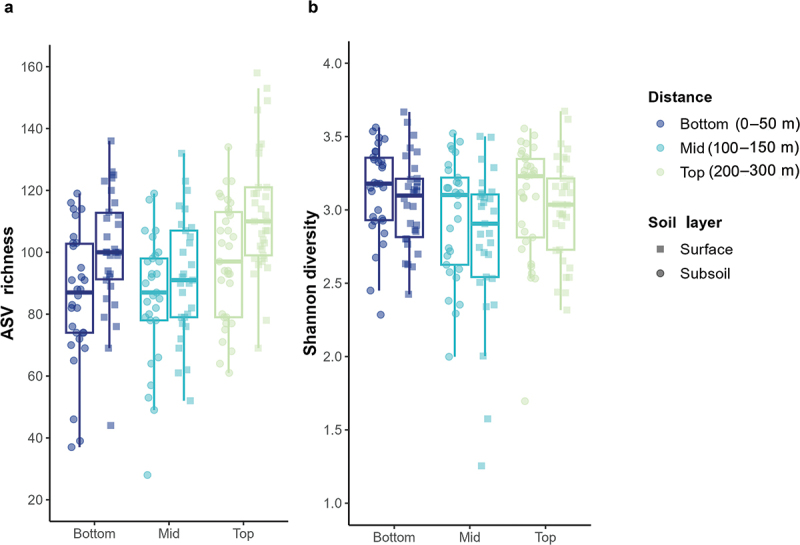
Alpha diversity indices for Robinson Ridge fungal soil communities, by transect distance group and soil layer. (a) Fungal observed richness (ASV counts). The Top group was significantly (*p* < 0.05) different to both the Mid and Bottom of transects. No significant difference in richness was found between Surface and Subsoils. (b) Shannon diversity index. Significant differences (*p* < 0.05) in diversity were observed between the Bottom and Top groups. There was no significant difference in diversity between Surface and Subsoils.

For diversity, the average Shannon indices for the distance groups were 3.1 (“Bottom”), 2.9 (“Mid”), and 3.0 (“Top”) (Figure 2). A significant difference (*p* < 0.05) was found between the “Bottom” and “Top” groups but there were no significant differences in diversity between the “Bottom” and “Mid” of transects (*p* > 0.06), or the “Mid” and “Top” (*p* > 0.3). There was also no significant (*F* = 0.14, *p* > 0.7) difference in diversity between Surface and Subsoils, both having an average value of 3.0.

**Figure 3.**
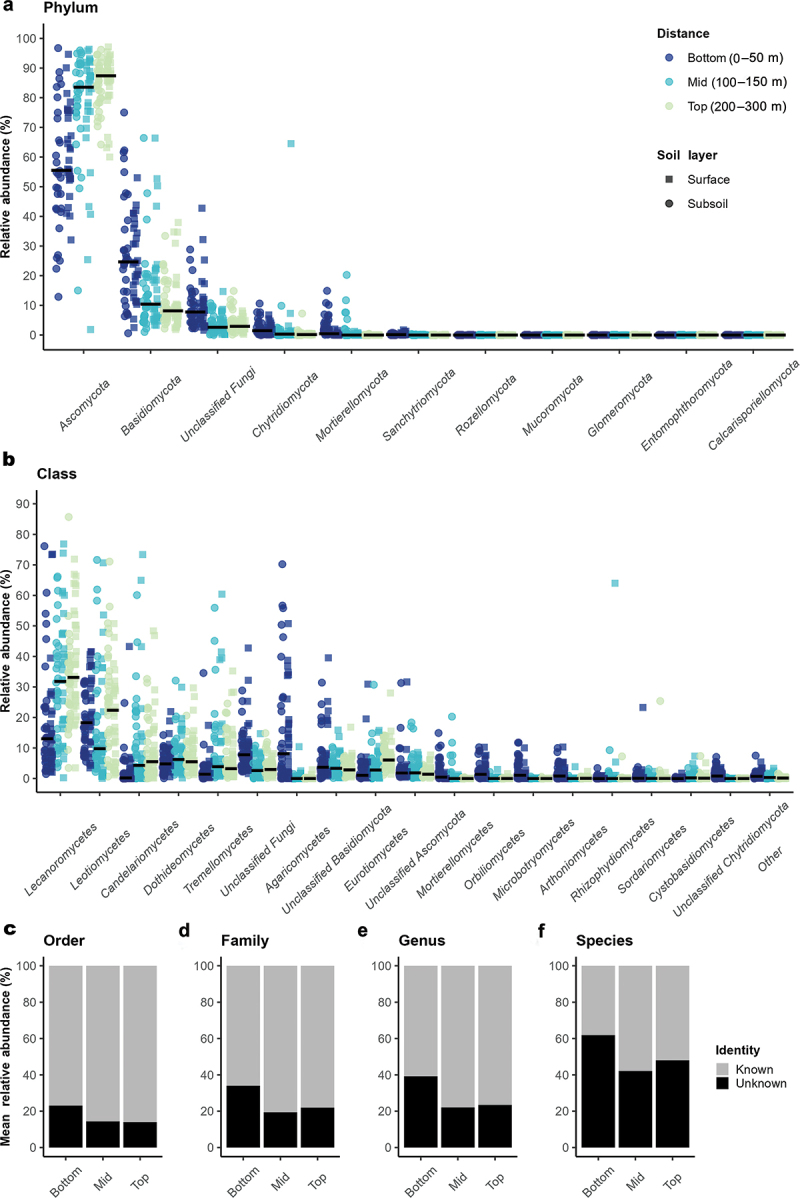
Relative abundances of fungal taxa, observed from ITS gene metabarcoding for 186 Robinson Ridge soils. Samples are visualised according to location on transect, grouped by Bottom (0–50 m), Mid (100–150 m), and Top (200–300 m), as well as soil layer (Subsoil or Surface). (a) Phylum level. Bars indicate median abundances for Subsoils (black) and Surface soils (grey). (b) Class level, showing the 18 most abundant classes. Bars indicate median abundances for Subsoils (black) and Surface soils (grey). (c–f) Mean relative abundances of identifiable (known) and unidentifiable (unknown) taxa at order, family, genus, and species levels, respectively.

The soil community was comprised of sequences representing 10 different fungal phyla. *Ascomycota* was the most abundant phylum overall, with a mean relative abundance of 73.6% (Figure 3a; Figure S1). *Ascomycota* showed the greatest abundance at the top of the transects (84.8%), compared with 58.6% and 77.4% for the “Bottom” and “Mid” groups, respectively. Other phyla were present in lower relative abundances, comprising *Basidiomycota* (18.1%), *Chytridiomycota* (1.5%), *Mortierellomycota* (1.0%), *Sanchytriomycota* (0.1%), *Rozellomycota* (0.001%), *Mucoromycota* (0.0005%), *Glomeromycota* (0.0001%), *Entomophthoromycota* (0.00001%), and *Calcarisporiellomycota* (0.00001%). The lowest median abundance of *Ascomycota* was found at the Bottom of transects (55.5%), compared with 83.5% and 87.3% at the “Mid” and “Top” of transects, respectively (Figure 3a; Figure S1). Correspondingly, the “Bottom” group contained higher median abundance of *Basidiomycota* (median 24.7%) compared with the “Mid” (10.4%) and “Top” (8.2%) groups, as well as for the lower abundance phyla such as *Chytridiomycota*, *Mortierellomycota*, and *Sanchytriomycota* (Figure 3a; Figure S1).

At the class level, over 40 classes were registered, within *Ascomycota* classes *Lecanoromycetes* and *Leotiomycetes* showed the greatest mean relative abundance overall, averaging 28.7% and 20.0%, respectively (Figure 3b; Figure S2). However, the range in abundances observed for these classes was pronounced, with *Lecanoromycetes* ranging from 0.2% to 85.7%, and *Leotiomycetes* from 0.06% to 71.6% (Figure 3b; Figure S2). Samples located at the “Mid” and “Top” of the transects showed higher median abundance of *Lecanoromycetes* (31.8% and 33.1% respectively) than those from the bottom (13.0%), while for *Leotiomycetes*, the highest median abundance was found in the “Top” group 22.4%, compared with 18.3% for the “Bottom” and 9.8% for the “Mid” groups (Figure 3b; Figure S2). The next four most abundant classes were the *Candelariomycetes* (7.9%), *Dothideomycetes* (7.3%), *Tremellomycetes* (6.8%), and *Eurotiomycetes* (4.7%). These three classes showed the greatest median abundances at the “Mid” or “Top” of transects (Figure 3b; Figure S2). Samples from the “Bottom” area of the transects, however, also showed high median abundances for classes such as *Agaricomycetes*, *Orbiliomycetes, Microbotryomycetes*, *Mortierellomycetes*, and *Arthoniomycetes* (Figure 3b; Figure S2).

Overall, many of the ITS sequences from the Robinson Ridge soil communities could not be classified as similar to known fungal taxa. For instance, at the phylum level, mean relative abundances of unclassified fungi amounted to 5.8%, and at the class level, 14% (Figure 3). At the lower taxonomic levels of order, family, genus and species, sequences with unknown classification amounted to 17%, 25%, 28%, and 51% respectively (Figure 3c–f). On average, soils at the “Bottom” of the transects contained higher proportions of unknown taxa compared with those from the “Mid” and “Top” distances (Figure 3c–e). This difference was greatest at the genus level, with 39% abundance of unknown taxa for the “Bottom”, compared with 22% and 23% for the “Mid” and “Top” of transect samples (Figure 3d).

### 3.2. Landscape heterogeneity in soil physicochemical properties

In the previous study of Wong et al. (2025) the soil physicochemical composition of Robinson Ridge showed to vary significantly among transect groups (*p* < 0.05). Soils at the “Bottom” of the slope, near moss beds, presented higher soil organic matter (mean = 4.17 ± 1.71%) compared to elevated sites (mean = 1.87 ± 0.71%). “Bottom” soils also exhibited the highest moisture content (mean = 8.41 ± 3.29%) and cation exchange capacity (eCEC) (mean = 2.77 ± 0.74 meq/100 g), indicating better nutrient-holding capacity. Additionally, they had higher electrical conductivity and higher concentrations of elements like total carbon, nitrogen, phosphorus, and sulphur. However, soil pH levels showed no significant differences across groups, ranging from 4.4 to 6.6 (Wong et al. 2025).

### 3.3. Relationships between fungal communities and environmental parameters

**Figure 4.**
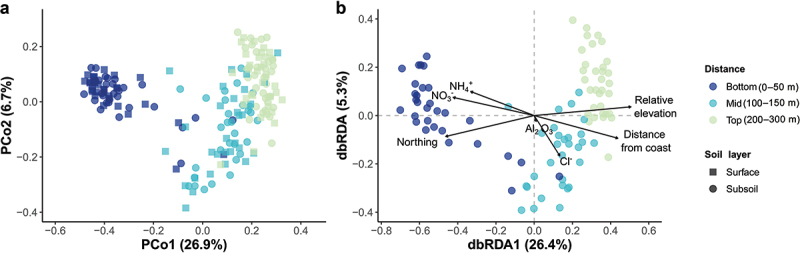
Relationships between fungal communities and edaphic parameters in Robinson Ridge soils. (a) Principal coordinates analysis of 186 Surface and Subsoil samples and (b) Distance-based redundancy analysis (dbRDA) for 93 Robinson Ridge Subsoils with significantly correlated edaphic parameters.

In multivariate analyses, clustering was observed according to locations of sample collection along transects, with the “Mid” and “Top” communities showing greater similarity to each other than to communities at the “Bottom” of the transects. Differences between distance groupings were statistically significant (*F* = 34.4, *p* = 0.001) (Figure 4). This clustering was consistent across both Subsoil and Surface communities, with statistically significant differences by distance group observed for both (Subsoils *F* = 17.52, *p* = 0.001; Surface soils *F* = 18.9, *p* = 0.001). In the dbRDA analysis, a total of 29.1% of the cumulative variation in fungal community composition could be explained by edaphic parameters (Table S1). The first and second axes explained 26.4% and 5.3% of the cumulative variation respectively (Figure 4b). Significant relationships were observed between the fungal community and seven edaphic parameters, with relative elevation most strongly correlated (*F* = 36.1, *p* = 0.001), followed by distance from the coast (*F* = 6.6, *p* = 0.001), NH_3_ (*F* = 3.1, *p* = 0.001), NO_3_^−^ (*F* = 3.3, *p* = 0.004), Al_2_O_3_ (*F* = 2.6, *p* = 0.001), and Northing (*F* = 1.8, *p* = 0.04) (Figure 4b).

**Figure 5.**
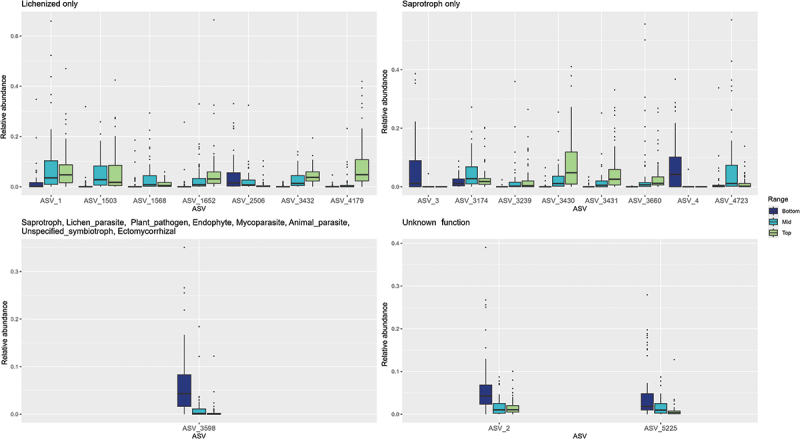
Relative abundance of the 18 most abundant ASVs per sample and their classification in terms of functionality, based on the FungalTraits database. Most of the ASVs were recognized as lichenized fungi only; three as saprotrophs only; and the others presented more than one option of functionality. Kruskal-Wallis test showed that ASVs related to lichenized taxa were significantly correlated to location (*p* < 0.05). ASV_2506 was recognized as g__*Caliciaceae*_gen_Incertae_sedis; ASV_1 as *Candelariella*, ASV_3 and ASV_4 as *Clitopilus*, ASV_3430 and ASV_3431 as *Cyathicula*, ASV_3660 as *Infundichalara*, ASV_1503 and ASV_1652 as *Lecidella*, ASV_3432 as *Micarea*, ASV_3239 as *Naganishia*, ASV_3174 as *Rachicladosporium*, ASV_4179 as *Sagedia*, ASV_1568 as *Shackletonia*, ASV_4723 as *Vishniacozyma*, ASV_2 as unclassified *Fungi*, ASV_3598 as unclassified *Helotiales*, ASV_5225 as unclassified *Basidiomycota*.

On closer examination, a group of ASVs were overrepresented in the Robinson Ridge soil, with eighteen ASVs accounting for 52.4% of all read counts (Figure 5). These were identified as belonging to *Candelariella*, *Clitopilus*, *Lecidella*, *Rachicladosporium*, *Nagashia*, *Cyathicula*, *Micarea*, *Infundichalara*, and representatives of unclassified *Helotiales*, *Caliciaceae*, and *Fungi* (Figure 5). These taxa have a range of common biotic interactions; most were designated as being lichenised and saprotrophs. A number of ASVs could not have their taxonomy at the genus level assigned, therefore we listed all the possible functions based on the available information for genera in those families. The other proposed functionalities were “lichen parasite”, “plant pathogen”, “endophyte”, “mycoparasite”, “animal parasite”, “unspecified symbiotroph”, and “ectomycorrhizal”. The 18 dominant ASVs also dominated the “Mid” and “Top” portions of the transect, and the seven ASVs corresponding to lichenised taxa were more abundant in the “Mid” and “Top” portions of the transect, accounting for 26% and 30% respectively, compared with only 8% for samples located at the “Bottom” of transects.

When considering the whole community, the two most common functional guilds were indeed “lichenised” and “saprotrophs”, specifically litter and soil saprotrophs (Figure S3). Other assigned functionalities were “algae parasite”, “animal parasite”, “arbuscular mycorrhizal”, “ectomycorrhizal”, “epiphyte”, “foliar endophyte”, “lichen parasite”, “mycoparasite”, also “plant pathogen”, “root endophyte”, and “sooty mold” (Figure S3). The proportion of ASVs that couldn’t have their functionality assigned was high, either due to limitations in assigning taxonomy or to missing information on FungalTraits database. At the “Bottom” portion of the transect, 52% of the fungi had functions that remain unidentified, against 26% at the “Mid” portion and 24% at the “Top” portion. Lichenised fungi represented 9% of the community at the “Bottom” portion of the transect, 35% at the “Mid” portion, and 38% at the “Top” portion. Litter saprotroph fungi represented 19% of the community at the “Bottom”, 16% at the “Mid” and 26% at the “Top”. Soil saprotrophs were present at the “Bottom” in the proportion of 5.0%, 11% at the “Mid”, and 3.0% at the “Top”. Kruskal-Wallis testing using the found functional classifications confirmed that the presence of lichenised fungi and soil saprotrophs was significantly correlated to location on the transect (*p* < 0.05). Spearman correlations showed a significant and negative correlation of lichenised fungi with nitrogen-relevant chemical variables and total carbon, with the strongest positive correlations being with dry matter fraction, PO_4_^3-^, relative elevation, and distance from the beginning of the transect (*p* < 0.05, Figure S4). The forward selection of environmental variables selected two significant variables PO_4_^3-^ and relative elevation. The amount of variance explained by the explanatory variables in the model was 23.48% (Figure S5).

### 3.4. Fungal strains isolated from Robinson Ridge soil

**Figure 6.**
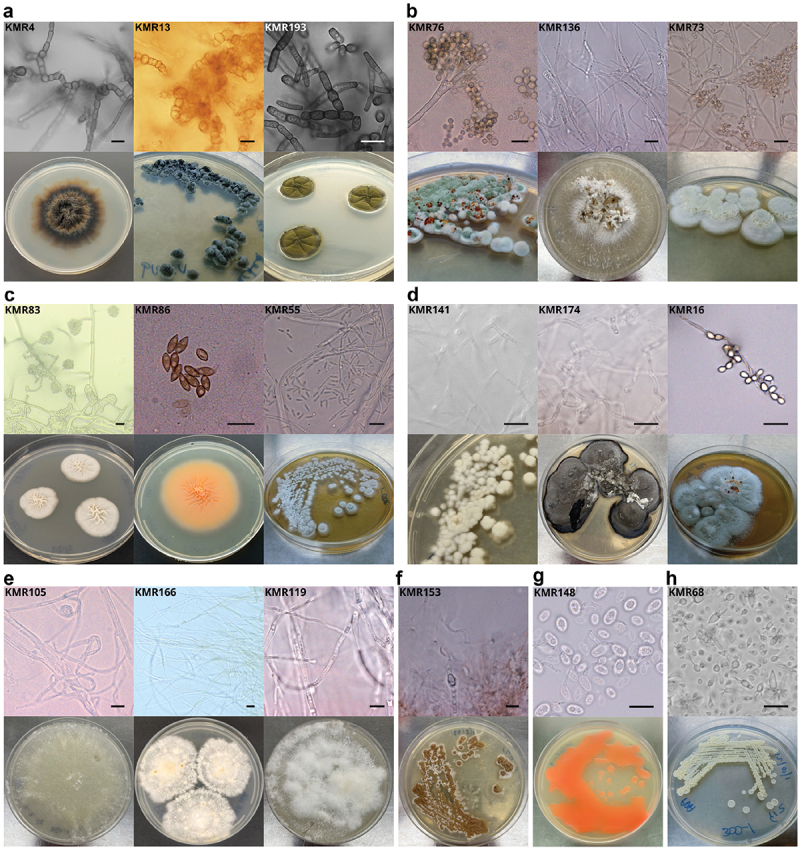
Colony morphology on potato dextrose agar and light microscopy for a selection of fungal strains (codes refer to the “Isolate number” as in Table 1) isolated from Robinson Ridge soil. Scale bars represent 10 µm. From the *Ascomycota*, classes included the (a) *Dothideomycetes*, characterized by dark pigmented colonies, slow growth and dark pigmented thick-walled arthroconidia and hyphae, (b) *Eurotiomycetes* characterized by light coloured colonies, hyaline hyphae and conidia produced abundantly from simple or compound conidiophores typical of the genera *Penicillium* and *Aspergillus*, (c) *Sordariomycetes* characterized by relatively fast growing colonies that can be variously pigmented and thin-walled conidia that are often hyaline, and (d) *Leotiomycetes* that are morphologically and ecologically diverse with variable colony and conidial morphology; while from the *Basidiomycota*, classes included the (e) *Agaricomycetes* characterized by rapid mycelial growth to produce abundant colonies with non-pigmented mycelium and no obvious spores, (f) *Exobasidiomycetes* characterized by yeast-like colonies morphology and simple single-celled propagules, (g) *Microbotryomycetes* characterized by yeast-like highly pigmented growth, and (h) *Tremellomycetes* again characterized by yeast-like growth on agar.

A total of 130 pure fungal isolates were recovered from FACS sorted cells when cultured on 0.1× RAVAN media (Table 1, Figure 6). Different tools were used to assign taxonomy by comparing two databases: the UNITE database and the SH-matching method, with variations between the two approaches observed. The first method identified sequences with the highest similarity to our own, while the SH-matching method employed a “species hypothesis” approach, using clustering algorithms to group sequences into candidate species based on genetic similarity (Kõljalg et al. 2013). The SH-matching method verified the presence of chimeras through VSEARCH (–usearch_global) chimera detection, and three samples were identified as potential chimeras, they were KMR7, KMR106 and KMR116, and each exhibited high similarity with sequences from GenBank database.

By consensus, we report that cultured isolates primarily belonged to the *Ascomycota* phylum (72%, *n* = 91, according to Species Hypothesis Matching), followed by *Basidiomycota* (24%, *n* = 31, according to Species Hypothesis Matching). Within the *Ascomycota*, isolates representing four classes were recovered: *Eurotiomycetes* (31%, *n* = 41) (Figure 6a), *Dothideomycetes* (24%, *n* = 31) (Figure 6b), *Sordariomycetes* (9%, *n* = 12) (Figure 6c), *Leotiomycetes* (3%, *n* = 4) (Figure 6d). Within *Basidiomycota*, five classes: *Agaricomycetes* (18%, *n* = 23) (Figure 6e), *Exobasidiomycetes* (4%, *n* = 5) (Figure 6f), *Microbotryomycetes* (0.8%, *n* = 1) (Figure 6g), *Tremellomycetes* (0.8%, *n* = 1) (Figure 6h), and *Cystobasidiomycetes* (0.8%, *n* = 1). Percentage similarity matches to known fungal species from the UNITE database ranged from 84.1%–100% (Table 1). The most common isolated genera were *Penicillium*, followed by *Cladosporium* and *Aspergillus*. Five isolates had no similar recognised matches for even phylum level in SH matching analysis, and may represent new taxa, e.g. KMR34, KMR83, KMR19, KMR182, and KMR67. Other three isolates had no close matches below the phylum level and 21 below the class level. At lower levels, 75% of all isolates had their identification at the genus level assigned, suggesting that a high proportion of new genera were also recovered.

### 3.5. Comparisons between cultured and environmental community fungal diversity?

**Figure 7.**
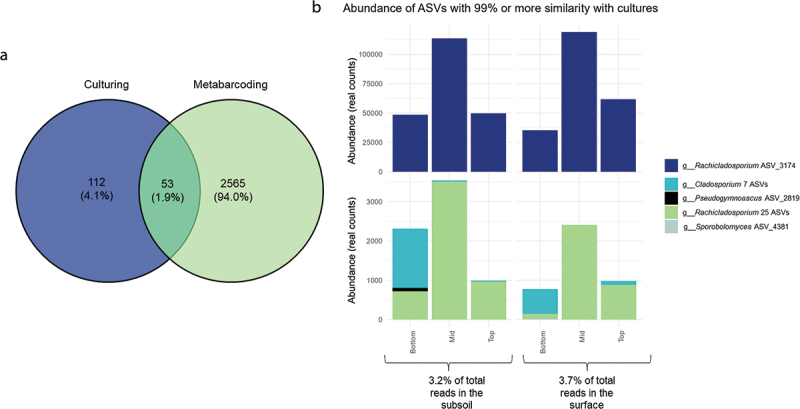
Comparisons between cultured and environmental community fungal diversity. (a) Venn diagram illustrating sequence overlap, based on blastn output. Culturing versus metabarcoding techniques shared only 1.9% of the total produced sequences. (b) Abundance of the 35 ASVs that matched isolate sequences. The barplot at the top shows only ASV_3174 identified as *Rachicladosporium*, while the barplot at the bottom shows the abundance of ASVs identified as *Cladosporium*, other *Rachicladosporium*, *Pseudogymnoascus*, and *Sporobolomyces*.

A large proportion of fungal ASVs were not shared between culture-dependent and culture-independent techniques (Figure 7). Culturing versus amplicon sequencing techniques shared only 1.9% of the total produced sequences (Figure 7a). Blastn results showed that 35 ASVs showed > 99% similarity with 18 different sequences from cultures. The 35 ASVs that matched isolate sequences belonged in the majority to the phylum *Ascomycota* (34 ASVs), with only one ASV belonging to *Basidiomycota*. They were identified as *Rachicladosporium*, *Cladosporium, Pseudogymnoascus*, and *Sporobolomyces*. Together, these units represented 3.2% of the total counts in the Subsoil and 3.7% of the total counts on the Surface (Figure 7b). Of these, *Rachicladosporium* ASV_3174 showed much higher abundance in comparison with all the others, representing 3.3% of the total number of counts alone. This ASV matches isolate KMR193 sequence with 100% sequence identity.

## 4. Discussion

The Windmill Islands region of East Antarctica is experiencing the effects of climate change (Robinson et al. 2018), and while extensive knowledge exists on moss beds, there is a notable gap in understanding their fungal communities. Previous studies on microbial community composition in Robinson Ridge revealed a substantial presence of fungi within the eukaryotic soil communities (Zhang et al. 2020; Wong et al. 2025) and also tracked their responses to changes in soils (Siciliano et al. 2014), but an in-depth investigation was still missing. Here, we assessed comprehensive fungal diversity, environmental heterogeneity at landscape scales, and gained new understanding of functional guilds present across the site. Greater proportions of lichenised fungi were observed at the top of the transect in lower moisture soils away from the coast. Novel culturing resulted in 130 fungi isolates, with 25% representing potential new genera, including isolates with as low as only 84.1% similarity with their top hits, and five isolates that could not be assigned at the phylum level. One hundred and twelve representative sequences were obtained exclusively through culture-dependent techniques, while 2,565 ASVs were obtained exclusively through culture-independent methods - with the number of representatives common to both approaches being low (1.9%) and in line with other authors’ suggestions (Figure 7; Vartoukian et al. 2010; Solden et al. 2016). The proportion of ASVs which were unable to be assigned at the genus level, as well as the high number of strains with no close relatives at the genus level, reinforces the argument that Antarctic environments harbour new and/or previously unreported fungal diversity (de Menezes et al. 2022).

### 4.1. Microbial community composition, diversity, and drivers at landscape scales

We found a clear spatial separation of fungal communities at the local landscape scale across Robinson Ridge (Figure 4) compared to eukaryotic communities’ data produced previously (Wong et al. 2025). Species richness was higher in the “Bottom” and “Top” areas of the transect, while diversity was higher at the “Bottom” (Figure 2), suggesting a more balanced community with a more even distribution of taxa abundances, likely due to favourable conditions of higher moisture, nutrient availability, and organic matter accumulation (Wong et al. 2025). Additionally, fungi at lower elevations appeared to prefer soils with high levels of NH_3_, NO_3_^−^, while fungi at higher elevations preferred higher Al_2_O_3_ and chloride (Figure 4).

As in previous studies of Antarctic soil communities, *Ascomycota* and *Basidiomycota* dominated (Cox et al. 2016; Ji et al. 2016). The most abundant groups belonged to two classes: *Lecanoromycetes*, a lichen-forming fungal class, and *Leotiomycetes*. These classes accounted for ten of the 18 most abundant ASVs and were found primarily at the “Mid” and “Top” regions of the transect. Lichenised fungi species were the most common functional group identified in the soil community, together with saprotrophic fungi (Figure 5) – a pattern also described for other Antarctic soil and cryptoendolithic communities (Coleine et al. 2018; Canini et al. 2020, 2021a). A comprehensive study assessing 200 archived Antarctic soils, from Livingston Island on the Antarctic Peninsula to Cape Hallett in northern Victoria Land showed a similar pattern in terms of dominant phyla which was also dominated by the class *Lecanorales* (composed almost completely by lichenised fungi; Dragone et al. 2025).

A study conducted across eleven ice-free areas of maritime Antarctica revealed that fungal assemblages have strong habitat specificity driven by both current environmental conditions (such as physicochemical properties, nutrient availability, and antagonistic interactions) and historical factors like colonisation potential (Zhang et al. 2022). The study also pointed to micro-niche availability within vegetation types, defending the dominance of lichens in drier zones, and the strong influence of other host types. This information aligns well with another finding of our study, the higher levels of *Leotiomycetes* in the “Mid” and “Bottom” areas of the transect. This taxon was reported as the most abundant in a study focusing on bryophyte covers in the Icelandic Highlands (Ortiz-Rivero et al. 2023), which may reflect the strong ecological association between this fungal lineage and plants, and converges with the dominance of mosses at the “Bottom” areas of our transect.

The two variables used in the RDA explained 23.48% of the variation in the functional community. This suggests that while these variables do explain some structure in the data, most of the variation remains unexplained. The strong correlation with relative elevation reinforces the highest presence of lichenised fungi in the “Mid” and “Top” regions of the transect. This finding aligns well with previous observations reporting a high abundance of bryophytes in soils with relatively high moisture at the “Bottom” of the transect, and a higher abundance of lichens in the relatively dry elevated areas (Ferrari et al. 2016; Wong et al. 2025). Phosphate may be a strong driver of the fungal distributions at Robinson Ridge due to the fact that phosphorous is a limiting element for lichens development (Souza Silva et al. 2025). Many filamentous fungi and yeasts isolated from Antarctic lichens can solubilise phosphate (Silva et al. 2022; Souza Silva et al. 2025). Thus, this ability may be related to the need of lichens to access mineral nutrients from rocks and soil (Silva et al. 2022). Another possible way to access this chemical could be the association of fungi with solubilising bacteria in the so-called “lichenic microbiome” (Silva et al. 2021).

Lichens are one of the first colonisers of terrestrial habitats; being able to survive in unusual environments, partially due to the existing self-supporting mutualism (Molnár and Farkas 2010). This relationship is known to have a certain level of specificity (Domaschke et al. 2012), also resulting in the production of a diverse array of secondary metabolites that are unique to lichen-forming fungi (Molnár and Farkas 2010). These compounds can protect the thalli from herbivores, pathogens, and competitors while also shielding them from external stressors such as intense UV radiation (Molnár and Farkas 2010). Many of these metabolites exhibit multiple biological activities, further contributing to the resilience and ecological success of lichens (Molnár and Farkas 2010).

“Mid” and “Bottom” areas also exhibited higher levels of *Leotiomycetes*, a taxon reported as the most abundant in a study focused on bryophyte covers in the Icelandic Highlands (Ortiz-Rivero et al. 2023), a finding that may reflect the strong ecological association between this fungal lineage and plants. *Leotiomycetes* includes numerous pathogenic, endophytic, saprophytic, and symbiotic species. Other identifiable fungal classes in the environmental data were *Candelariomycetes* (formerly placed within *Lecanoromycetes*) (Voglmayr et al. 2019), *Tremellomycetes*, and *Eurotiomycetes*, which were also more abundant in the “Mid” and “Top” sections of the transects. *Tremellomycetes*, and *Eurotiomycetes* are known for their psychrophilic or psychrotolerant adaptations (Canini et al. 2021b). Another abundant class, *Dothideomycetes*, was particularly dominant in the “Mid” region samples. Together with *Eurotiomycetes*, these fungi include members classified as “black fungi”; melanised species capable of colonising extreme environments due to their resistance to desiccation and UV exposure (Selbmann et al. 2005; Ruibal et al. 2009). Their presence may also play a crucial role in shielding other microbes within the same community from excessive UV radiation (Choe et al. 2018).

Other taxa which were significantly more abundant at the “Bottom” of the transects included unclassified *Agaricomycetes*, *Orbiliomycetes*, *Microbotryomycetes*, *Mortierellomycetes*, and *Arthoniomycetes*. Within these, *Agaricomycetes* showed a higher abundance. This taxon has been previously detected living in symbiosis with mosses (Ortiz-Rivero et al. 2023); relationships like this could be driven by nutrient alterations, including nitrogen and microelement deficiencies, alongside high organic, uronic, and aromatic acid content in exudates (Kachalkin et al. 2008). For example, dominant yeasts in moss turf have a broad assimilation spectrum, enabling them to utilise organic acids and aromatic compounds (Kachalkin et al. 2008; Ortiz-Rivero et al. 2023). These observations are in accordance with the higher levels of moisture, nutrients, organic matter accumulation in the transect’s “Bottom” soils described by Wong et al. (2025).

### 4.2. Novel approaches to isolate fungal dark matter from Antarctic soil

We focused our study on the isolation of slow growing fungi, as microbial growth under oligotrophic, cold conditions may allow rare organisms to persist without being outcompeted by faster-growing species (Gostinčar et al. 2022). The initial inoculation of soil in water supplemented with hydrogen gas was based on prior empirical observations in our laboratory, originally aimed at the isolation of high-affinity hydrogen-oxidising bacteria (unpublished data). Interestingly, this method appeared to favour the proliferation of slow-growing fungal taxa. One possible explanation is that the inoculation in water may have reduced the biomass of dominant fungi, opening ecological space for rare oligotrophic species, particularly those that thrive in nutrient poor, low bacterial biomass environments. However, this remains a hypothesis rather than a validated mechanism, and for that reason, we chose not to offer a definitive explanation for the unexpectedly high diversity of isolated fungi. Other factors may also account for this outcome; for instance, hydrogen gas itself might provide some benefit to fungal growth, as suggested by Zhang et al. (2017).

Fungi have rarely been addressed in the more recent cultivability literature (Rämä and Quandt 2021). In our study, with a new technique integrating initial inoculation of soil in water supplemented with hydrogen gas, long incubation at low temperatures and fluorescence-activated cell sorting, we recovered 130 isolates from one soil sample with the proportion of *Ascomycota* obtained similar to that observed in the metabarcoding analysis. *Basidiomycota* was the second most abundant cultured phylum, but, unlike in the metabarcoding data, no other fungal phyla were represented among the isolates (Table 1). Overall, a high proportion of potential new genera were also recovered.

Within the covered classes, *Eurotiomycetes* and *Dothideomycetes* represented the largest portion of the total isolated taxa (Table 1). Members from these taxa exhibit strategies to survive in extreme Antarctic conditions, especially the highly melanised genera such as *Friedmanniomyces, Cryptococcus*, and *Cryomyces* (Coleine et al. 2024). These classes are known for their resilience to extreme temperatures, UV radiation, and efficiency in DNA repair and the degradation of pollutants. Thus, they are becoming a focal point of research across fields including microbial ecophysiology, evolutionary adaptations to extreme environments, geomycology, and applied research (Selbmann et al. 2014; Coleine et al. 2024). Reference genomes for black fungal lineages are still limited, therefore providing pure cultures could open up exciting opportunities, enabling in-depth investigation into their biotechnological, bioremediation, and radioprotection capabilities. The most common genera isolated within these classes were *Penicillium*, followed by *Cladosporium*, *Aspergillus*, and *Rhinocladiella*. The sample KMR99, for example, was recently covered in another study by Furnell et al. (2025), where the focus was the taxonomic description and recognition of a newly identified fungal species, *Penicillium psychrofluorescens*.

Despite the high abundance of *Lecanoromycetes* in the metabarcoding analysis, none of the cultivated isolates belonged to this class. This can easily be explained by the difficulties in culturing fungi with obligate symbiotic lifestyles (Coleine et al. 2018). The other two most abundant classes represented by cultivated strains were *Sordariomycetes* and *Agaricomycetes*. *Sordariomycetes* constitute the second largest class within *Ascomycota* (Chen et al. 2023) and have been shown to tolerate polycyclic aromatic hydrocarbons (PAHs) in studies of creosote-polluted soils (Aranda 2016). They also function as PAHs degraders in petroleum-contaminated soils, as shown in soils from Quebec, Canada (Marchand et al. 2017). Many members of this taxon can degrade organochlorides, pesticides, or aromatic hydrocarbons (Marco-Urrea et al. 2015). Additionally, studies on lichen-associated fungi in Antarctica have also identified *Sordariomycetes* representatives (Park et al. 2015). The fourth most represented class among the isolates was *Agaricomycetes* (*Basidiomycota*), which was not among the most abundant classes in our environmental data. This class is an infrequent taxon in Antarctic soils, but a dominant fungal taxon found in most soils of the world (Tedersoo et al. 2014; Cox et al. 2016). As this class commonly forms ectomycorrhizas with woody species at lower latitudes, the much lower abundance may be explained by the absence of woody plant material in the sampled environment, or by long-distance dispersal limitations (Cox et al. 2016).

### 4.3. Overlap between culture-dependent and culture-independent methods

Despite the high diversity raised by sequencing, it is important to observe that a significant portion of environmental DNA can belong to inactive, dormant, or even dead cells, taxa that may only become active for short periods or even taxa not truly capable of growing under Antarctic extreme conditions (Carini et al. 2016; Canini et al. 2024). This effect can be even more common in Antarctic soils due to the limited microbial activity and the combination of dry, cold, and saline conditions that contribute to a stabilising effect on DNA (Canini et al. 2024).

The overlap between culture-dependent and culture-independent techniques for assessing fungal diversity was minimal, being only 1.9%. The cultivated fraction represented 4.1% of the total representative sequences, while metabarcoding represented 94.0%. This outcome was expected, as only 0.1%–1% of microorganisms in an environmental sample can typically be recovered through cultivation (Vartoukian et al. 2010; Solden et al. 2016). Consequently, molecular methods have become a less biased and often more efficient approach for assessing fungal community diversity (Cazabonne et al. 2022). Despite the limited recovery of strains through cultivation, previous studies have demonstrated its importance in Antarctic soils. For instance, a study on aerobic anoxygenic phototrophic bacteria recovered 77 unique isolates, 47 of which were not detected by 16S rRNA gene amplicon sequencing of community DNA (Tahon and Willems 2017). Similarly, a genus-level comparison of cultivated heterotrophic bacteria from exposed Antarctic soils with 16S rRNA gene pyrosequencing data revealed that 25.6% of genera identified by cultivation were undetected by sequencing (Tytgat et al. 2014). The cultured library of 130 isolates retained here is valuable, as it enables the creation of genetic libraries that could later be used to analyse various genetic markers or undergo whole-genome sequencing for genomic and comparative genomic studies, potentially combined with (comparative) transcriptomics (Burgaud et al. 2022). This is often crucial for definitive species identification, enforcing the importance of culturing approaches.

The recovery of 2,600 ASVs through metabarcoding highlights the importance of complementing culture-based approaches with high-throughput DNA sequencing to gain deeper insights into microbial diversity, particularly for taxonomically unassigned groups. Notably, some sampled areas had more than half of their community composition unassigned at the genus level. Among the 35 ASVs that matched sequences from cultured isolates, ASV_3174 was by far the most abundant, accounting for 3.7% of the total counts. This ASV exhibited 100% sequence identity with the cultured isolate KMR193, which was identified as *Rachicladosporium* sp. Notably, four cold-adapted, endolithic *Rachicladosporium* species from Antarctica and the Italian Alps were recently reassigned to the newly established genus *Cryoendolithus* (Piątek et al. 2023).

Our study underscores the value of integrating culture-dependent and culture-independent methods to gain a more comprehensive understanding of fungal distribution, diversity, and functional potential. The documentation of unknown diversity highlights the significant presence of “fungal dark taxa” in Robinson Ridge and opens space for further investigations aiming at the recognition of these groups. The isolation of strains from groups known for producing important secondary metabolites, along with the high fungal diversity observed in regions with low moisture and nutrient availability, demonstrates the remarkable resilience of fungi to extreme environmental conditions such as prolonged desiccation and intense solar radiation. Moreover, the recovery of fungal classes, renowned for their secondary metabolite production, opens new discussions on the vast potential of Antarctic fungi for a wide range of industrial and medical applications.

## Supplementary Material

final-Supplementary_Material_nmml.docx

## Data Availability

Complete genomic data and services are available at the Australian Antarctic Data Centre under “AAS_4406_Robinson_Ridge_2019_DNA” (Wong and Ferrari 2025). ITS sequences have been submitted to NCBI under the submission number: SUB15364809, with the exception of the already published *Penicillium psychrofluorescens* KMR99 (OR797067.1).
