# Supplementary material for: Integrating novel culturing and culture‐independent methods to unveil soil fungal dark matter, functional guilds and environmental drivers across Robinson Ridge, East Antarctica
Source: Mycology. 2025 Dec 9;17(2):635–58. doi: 10.1080/21501203.2025.2579297 (PMC13267029; doi:10.1080/21501203.2025.2579297)
Supplement: final-Supplementary_Material_nmml.docx [file TMYC_A_2579297_SM4659.docx]

**Supplementary material**

**Table**

**Table S1.** Mean and standard deviation for seven significantly correlated environmental parameters by transect distance group.

| **Distance** | **Bottom (0–50 m)** | **Mid (100–150 m)** | **Top (200–300 m)** |
| --- | --- | --- | --- |
| Distance from coast (m) | 177.0 ± 9.0 | 208.5 ± 3.8 | 198.6 ± 2.1 |
| Relative elevation (m) | 1.2 ± 2.1 | 11.4 ± 1.7 | 21.0 ± 0.4 |
| Northing (m) | 2639030 ± 14.0 | 2638938 ± 15.0 | 2638832 ± 29.0 |
| Cl (ppm) | 290.0 ± 53.7 | 259.8 ± 53.2 | 300.3 ± 70.3 |
| Al_2_O_3_ (%) | 13.6 ± 0.5 | 13.7 ± 0.3 | 13.5 ± 0.3 |
| NH_3_ (mg/kg DMB) | 1.03 ± 0.6 | 0.3 ± 0.2 | 0.4 ± 0.3 |
| NO_3_^-^ (mg/kg DMB) | 7.4 ± 6.6 | 0.5 ± 0.3 | 0.5 ± 0.3 |

**Figures**


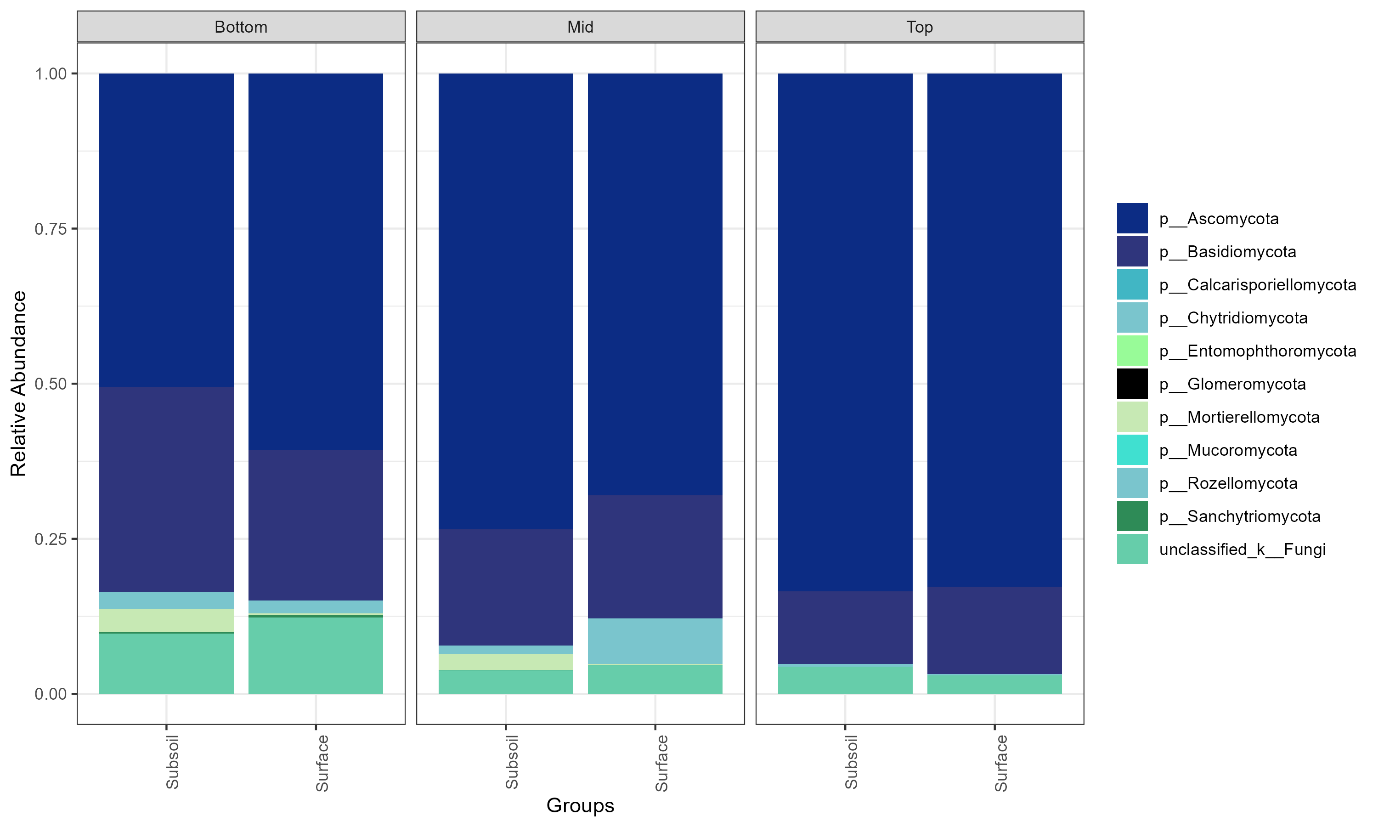


**Figure S1.** Bar plot Relative abundances of fungi at the phylum level, observed from ITS metabarcoding for 186 Robinson Ridge soils. Samples are visualized according to location on transect, grouped by “Bottom” (0–50 m), “Mid” (100–150 m), and “Top” (200–300 m), as well as soil layer (subsoil or surface).


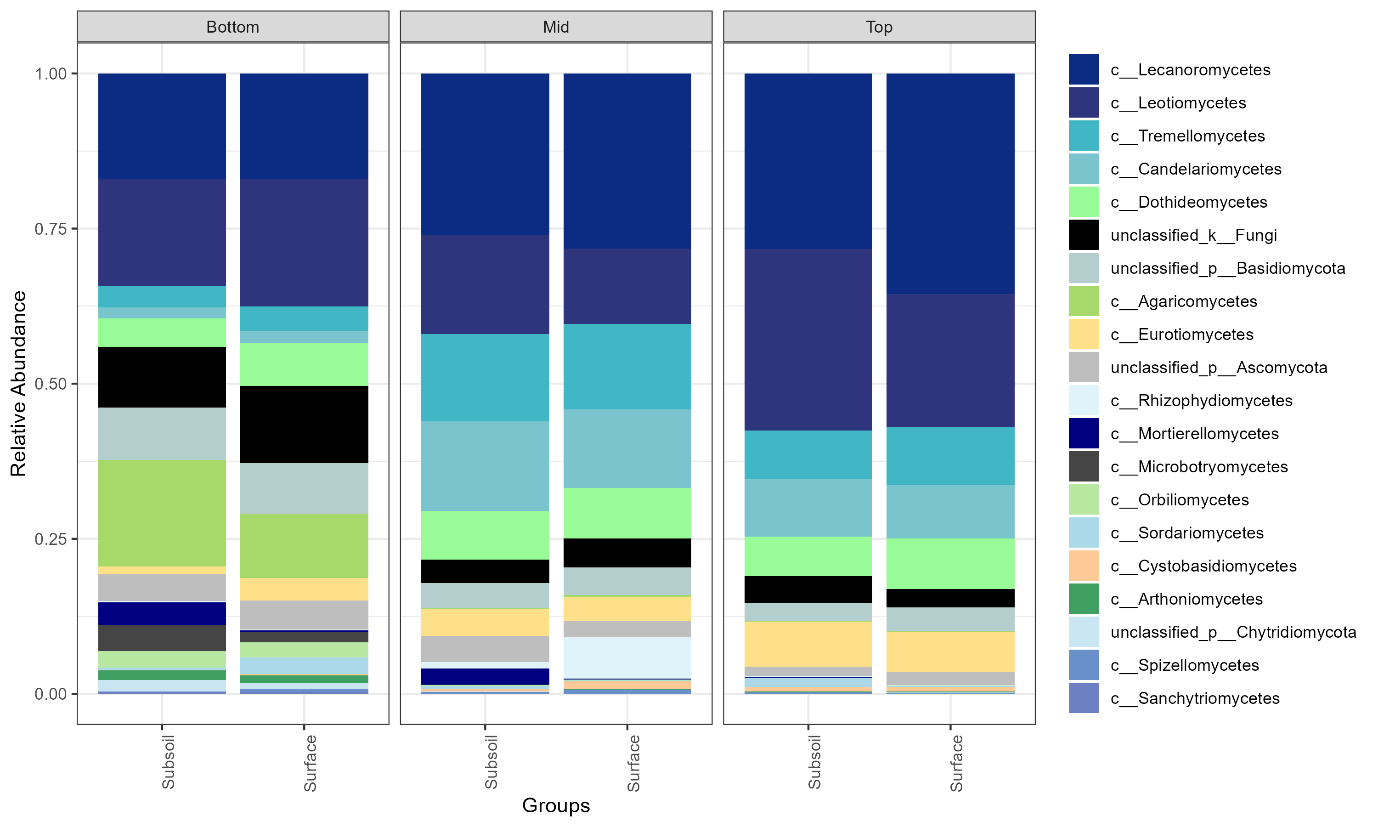


**Figure S2.** Bar plot Relative abundances of fungi at the class level showing the 20 top classes, observed from ITS metabarcoding sequencing for 186 Robinson Ridge soils. Samples are visualized according to location on transect, grouped by “Bottom” (0–50 m), “Mid” (100–150 m), and “Top” (200–300 m), as well as soil layer (subsoil or surface).

**
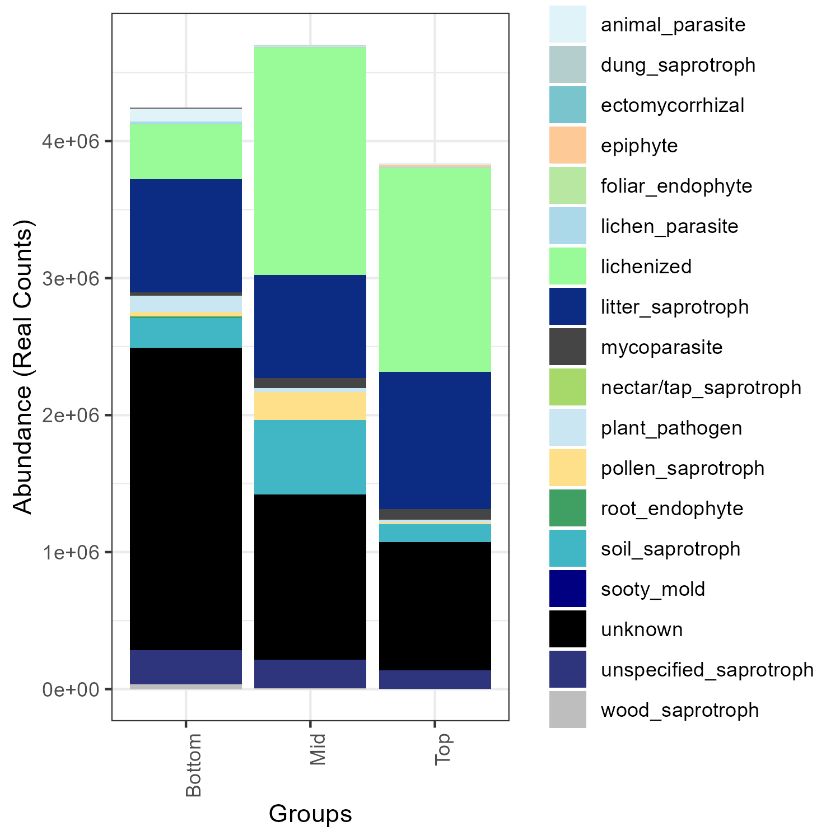
**

**Figure S3.** Bar plot showing the actual abundances of fungal functional guilds, categorized by primary lifestyle as defined by FungalTraits. Functional roles are displayed according to their location along the transect, grouped by “Bottom” (0–50 m), “Mid” (100–150 m), and “Top” (200–300 m).


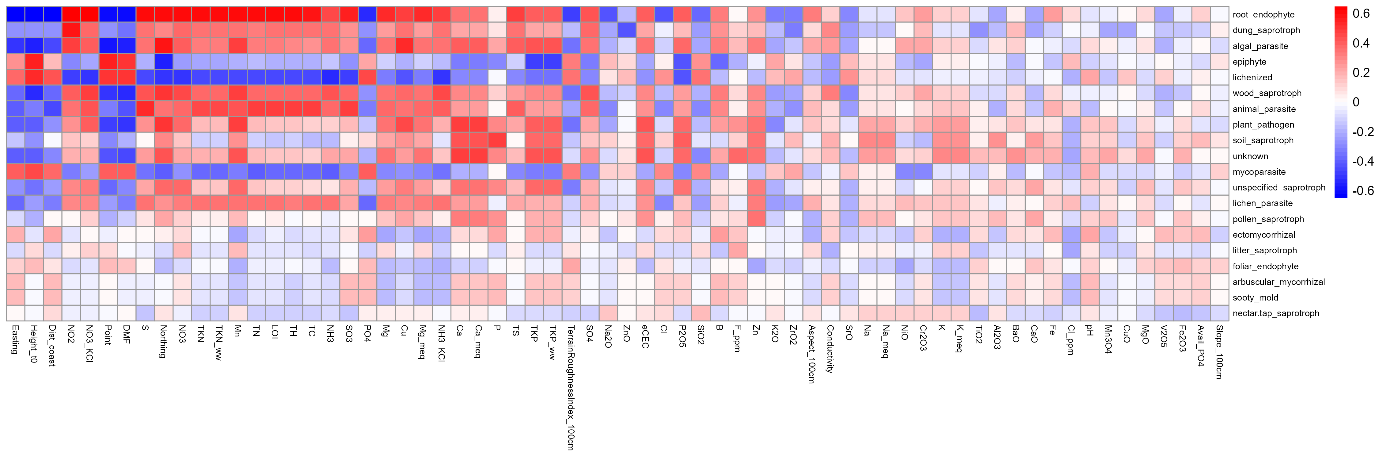


**Figure S4.** Heatmap of significant Spearman Correlations (FDR-adjusted p < 0.05) between fungi functionality and environmental variables.


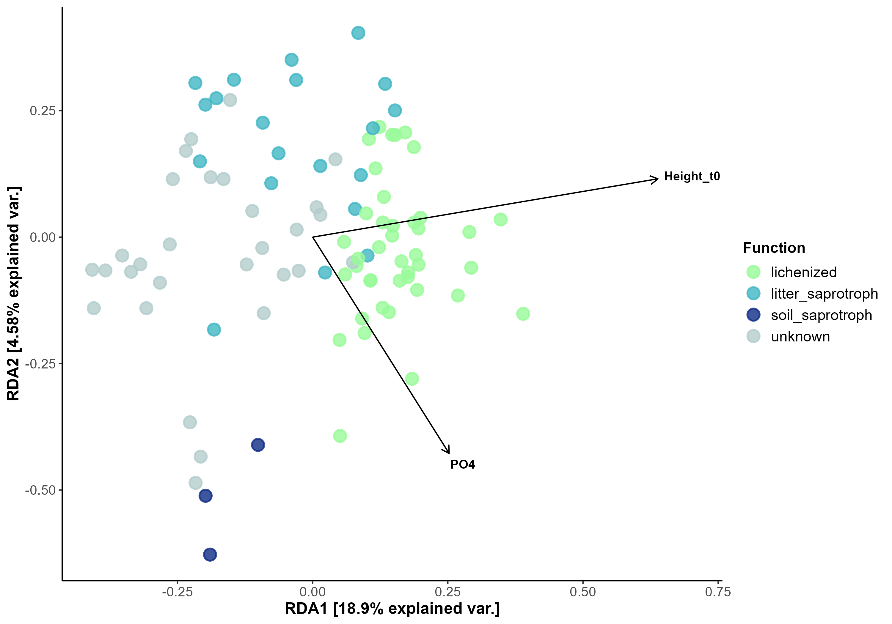


**Figure S5.** Redundancy analysis showing community variation explained by environmental variables. 23.48% of community variance explained by predictors.
